# Gold Nanodots‐Anchored Cobalt Ferrite Nanoflowers as Versatile Tumor Microenvironment Modulators for Reinforced Redox Dyshomeostasis

**DOI:** 10.1002/advs.202406683

**Published:** 2024-07-10

**Authors:** Guicheng Zeng, Jinning Mao, Haiyan Xing, Zhigang Xu, Zhong Cao, Yuejun Kang, Guodong Liu, Peng Xue

**Affiliations:** ^1^ School of Materials and Energy Southwest University Chongqing 400715 China; ^2^ Health Management Center The Second Affiliated Hospital of Chongqing Medical University Chongqing 400016 China; ^3^ School of Biomedical Engineering Shenzhen Campus of Sun Yat‐sen University Shenzhen Guangdong 518107 China; ^4^ Department of Neurosurgery The Second Affiliated Hospital of Chongqing Medical University Chongqing 400016 China

**Keywords:** cobalt ferrite, nanocatalysts, reactive oxygen species, sonodynamic therapy, tumor microenvironment

## Abstract

Given that tumor microenvironment (TME) exerts adverse impact on the therapeutic response and clinical outcome, robust TME modulators may significantly improve the curative effect and increase survival benefits of cancer patients. Here, Au nanodots‐anchored CoFe_2_O_4_ nanoflowers with PEGylation (CFAP) are developed to respond to TME cues, aiming to exacerbate redox dyshomeostasis for efficacious antineoplastic therapy under ultrasound (US) irradiation. After uptake by tumor cells, CFAP with glucose oxidase (GOx)‐like activity can facilitate glucose depletion and promote the production of H_2_O_2_. Multivalent elements of Co(II)/Co(III) and Fe(II)/Fe(III) in CFAP display strong Fenton‐like activity for·OH production from H_2_O_2_. On the other hand, energy band structure CFAP is superior for US‐actuated ^1^O_2_ generation, relying on the enhanced separation and retarded recombination of e^−^/h^+^ pairs. In addition, catalase‐mimic CFAP can react with cytosolic H_2_O_2_ to generate molecular oxygen, which may increase the product yields from O_2_‐consuming reactions, such as glucose oxidation and sonosensitization processes. Besides the massive production of reactive oxygen species, CFAP is also capable of exhausting glutathione to devastate intracellular redox balance. Severe immunogenic cell death and effective inhibition of solid tumor by CFAP demonstrates the clinical potency of such heterogeneous structure and may inspire more relevant designs for disease therapy.

## Graphical abstract

CFAP with CAT‐, POD‐, and GPx‐mimic activities is successfully developed as a versatile tumor microenvironment modulators for reinforced redox dyshomeostasis. CFAP acts as a qualified sonosensitizer, where Au decoration can facilitate depleting glucose and scavenging electrons during sonosensitization. Animal studies proved the potency of CFAP for high‐efficient tumor eradication.

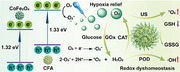

## 1 Introduction

Physiological redox balance profoundly impacts tumor initiation, differentiation, proliferation, metastasis, apoptosis, autophagy, and even cause therapeutic resistance to promote cancer development.^[^ ^1^, ^2^ ^]^ In the light of this, intelligent induction of redox dyshomeostasis holds special promise to inhibit tumor growth and spreading.^[^ ^3^ ^]^ The elaborate cooperation of reactive oxygen species (ROS) system and antioxidant defense (AOD) system contributes to the sustainable redox balance of tumor. Moderate endogenous generation of ROS can mediate cell signaling transduction for tumorigenesis, tumor promulgation and metastasis.^[^ ^4^, ^5^ ^]^ However, intracellular ROS level beyond its threshold value would cause irreversible destruction to proteins, lipids, and nucleic acids, which is regarded to be oxidative damage.^[^ ^6^, ^7^ ^]^ Endoplasmic reticulum (ER) stress and electron leakage from mitochondrial respiratory chain can amplify the cytosolic ROS content.^[^ ^8^ ^]^ Apart from these metabolic activities, stimulus‐responsive catalytic reactions can also enrich cytoplasm with excessive ROS.^[^ ^9^ ^]^ For instance, sonodynamic therapy (SDT) eliminates tumor cells through massive ROS production under ultrasound (US)‐triggered sonosensitization, with the merits of minimal invasiveness and maximum tissue‐penetration depth.^[^ ^10^, ^11^, ^12^, ^13^ ^]^ To date, many nano‐sized inorganic sonosensitizers, including TiO_2_, BiVO_4_, MnWO_x_, etc., as well as their affiliated heterojunctions and Schottky junctions, have been developed to convert the circumjacent H_2_O or O_2_ to cytotoxic ROS.^[^ ^14^, ^15^, ^16^, ^17^ ^]^ The structural design criteria of these high‐performance agents lie on the rapid separation and retarded recombination of e^−^/h^+^ pairs within the energy band.^[^ ^18^ ^]^ Alternatively, chemodynamic therapy (CDT) employs multivalent metal ion (e.g., Fe^2+/3+^, Cu^1+/2+^, Mn^2+/3+^, Co^2+/3+^) as Fenton agents to damage tumor cells, by converting endogenous H_2_O_2_ to hydroxyl radical (·OH).^[^ ^19^, ^20^, ^21^, ^22^ ^]^ Given the above facts, the combination of SDT and CDT via an integrative nanoagent may reinforce ROS generation to amplify the fatal oxidative damage.^[^ ^23^, ^24^ ^]^ On the other hand, AOD system can scavenge the excessive ROS to restore the kinetic equilibrium of cellular redox, and AOD disturbance is expected to aggravate redox dyshomeostasis to exacerbate cell injury.^[^ ^25^ ^]^ Glutathione (GSH) system is a major thiol antioxidant system, which can cycle in between its reduced and oxidized states depending on the oxidative status.^[^ ^26^ ^]^ GSH depletion can not only protect ROS from elimination but also trigger ferroptosis upon the accumulation of lipid peroxides.^[^ ^27^ ^]^ To this end, a rational design of steady nanoplatforms is recommended to promote redox dyshomeostasis by virtue of versatile operating mechanisms.

Cancer develops in complex and dynamic tumor microenvironment (TME), where supports tumor stemness, survival, proliferation, angiogenesis, invasion, and metastasis through complex signaling networks.^[^ ^28^, ^29^ ^]^ The aberrant compositions of TME produces mild acidic pH condition, severe hypoxia, high‐level H_2_O_2_, and GSH, etc.^[^ ^30^ ^]^ Tumor acidity is associated with metabolic reprogramming and the engagement of aerobic glycolysis, and lower pH can increase proteinase expression and metalloprotease activities, which aids the migration and invasion of tumor cells.^[^ ^31^ ^]^ Intra‐tumoral acute hypoxia is primarily caused by the inefficient blood supply from abnormal vascularization, which accelerates tumor progression by activating the signaling pathway of hypoxia‐inducible factors (HIF).^[^ ^32^ ^]^ The increase production of H_2_O_2_ occurs in dysfunctional mitochondria, which fertilizes the TME by chronic inflammation for agitating tumor recurrence and metastasis.^[^ ^33^ ^]^ Correspondingly, GSH level is concurrently raised to counteract the overexpressed H_2_O_2_, as a result of the operation of ROS defense mechanisms. Besides the pivotal roles of these TME cues in strengthening tumorigenesis, they also hamper many therapeutic modalities and weaken curative effects. For instance, hypoxia in TME always compromises the treatment efficacy of oxygen‐dependent procedures (e.g., SDT and tumor starvation by glucose exhaustion).^[^ ^34^, ^35^ ^]^ In addition, overabundance of GSH can foster oxidative stress resistance, which is detrimental to ROS‐relevant therapies. Provided by the importance of TME in expediting tumor development and therapy resistance, strategies that can modulate TME cues are ungently requisite to improve the curing efficacies. Qualified candidates are conceived to have the following three characteristics. First, they should be able to normalize or inversely regulate these TME cues through hypoxia reversal, acidity neutralization and lowering the grade of redox balance. Second, they can react with these abnormal TME factors to generate cytotoxic substances, resulting in multiple patterns of cell death. Third, they need to be inert to normal cells and have no influence on body metabolism. To this end, novel nanotechnology‐based approaches aiming at targeting and reprogramming aberrant TME are consistently being explored toward both fundamental research and practical applications.

**Scheme 1:**
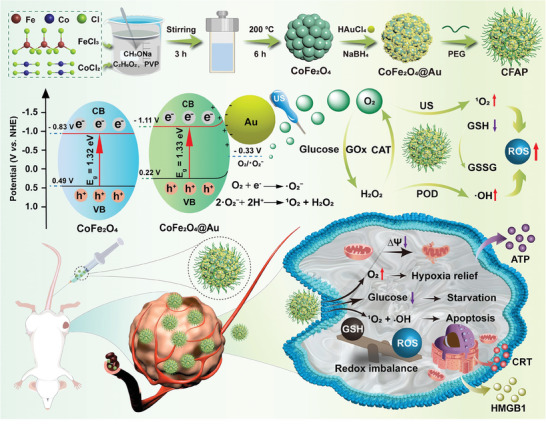
Diagram of the synthetic route of CFAP as TME modulator and its operating mechanism for inducing reinforced redox dyshomeostasis.

Cobalt ferrite (CoFe_2_O_4_), as a pronounced transition‐metal chalcogenide catalyst, takes part in renewable energy conversion and environmental pollutant clearance.^[^ ^36^, ^37^ ^]^ Moreover, both iron and cobalt are essential elements for human health, which are indispensable for production of hemoglobin and vitamin B_12_, respectively.^[^ ^38^, ^39^ ^]^ Taking the advantages of the rapid separation of e^−^/h^+^ pairs under US actuation, as well as the redox couples of Fe^2+^/Fe^3+^ and Co^2+^/Co^3+^ as Fenton agents, considerable ROS production by nano‐sized CoFe_2_O_4_ has been demonstrated by our group toward tumor cell killing.^[^ ^18^ ^]^ Nevertheless, the yield of ROS is still expected to be further amplified by delaying the recombination of e^−^/h^+^ pairs and accelerating charge transfer in Fenton reaction center through optimizing the energy band structure of CoFe_2_O_4_. Moreover, ROS‐induced oxidative stress alone is not sufficient to induce an irreversible cell death, which may further cause tumor recurrence or relapse after initial treatments. Taking account of the above circumstances, in this study, PEGylated gold (Au) nanodots‐anchored CoFe_2_O_4_ nanoflowers (CFAP) were developed for as versatile TME modulators, aiming at reinforced redox dyshomeostasis against tumor (**Scheme** **1**). The CoFe_2_O_4_@Au Schottky junction was synthesized through in situ nucleation and growth of Au nanodots onto the vicinal surface of CoFe_2_O_4_. The conjugated Au can serve as the electron scavenger to effectively retard the recombination of e^−^/h^+^ pairs, which significantly improves the charge utilization efficiency under US irradiation. In addition, the accelerated charge transfer during sonosensitization promotes Fenton‐like reactions mediated by the multivalent Fe^2+^/Fe^3+^ and Co^2+^/Co^3+^ redox couples. Such enhanced SDT and CDT are liable to produce abundant ROS for oxidative damage. Furthermore, CoFe_2_O_4_@Au possesses GSH peroxidase (GPx) activity, which can deplete intracellular GSH to aggravate redox imbalance. On the other hand, tiny Au nanodots act as glucose oxidase (GOx)‐mimic enzyme for tumor starvation through glucose deprivation, which can effectively synergize with redox dyshomeostasis therapy.^[^ ^40^, ^41^ ^]^ Importantly, the intrinsic catalase‐like activity of CoFe_2_O_4_@Au catalyzes the decomposition of H_2_O_2_ into O_2_, and the resultant hypoxia relief tends to accelerate glucose consumption and promotes SDT process. Therapy‐induced apoptosis/ferroptosis by CFAP arouses efficient immunogenetic cell death (ICD), which can stimulate antitumor immunity for systemic cancer therapy. Compared to the previous studies in CoFe_2_O_4_, the innovation points of CFAP can be briefly clarified to be the following points. First, CFAP provides a practical strategy to engineer cobalt ferrite, aiming at optimizing its energy band structure to acquire a promoted therapeutic efficacy. Second, CFAP enables other treatment modalities to synergize with the CoFe_2_O_4_‐induced redox dyshomeostasis, which is essential for rendering a complete tumor eradication. Third, CFAP is responsive to many TME cues and can thereby operate without harsh reaction conditions. The morphology, energy band structure and catalytic activities of final products were comprehensively characterized from various perspectives. Splendid therapeutic effects of CFAP were validated both at the cellular level and on BALB/c mice‐bearing 4T1 tumor models.

## 2 Results and Discussion

### 2.1 Structural Characterizations

**Figure 1:**
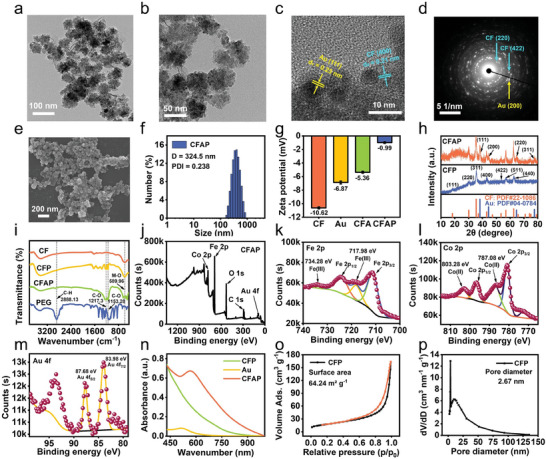
Structural characterizations. a) Low‐magnification and b) high‐magnification TEM images of CFAP. c) High‐resolution TEM image CFAP. The interplanar spacing of 0.21 and 0.23 nm represents the crystalline structure of CoFe_2_O_4_ and Au, respectively. d) Ring electron diffraction pattern of CFAP. e) FESEM image of CFAP. f) Hydrodynamic size of CFAP measured by DLS. g) Zeta potentials of CoFe_2_O_4_, Au, CFA and CFAP. h) XRD patterns of CFP and CFAP. i) FTIR spectra of PEG, CF, CFP and CFAP. j) Full‐survey XPS spectrum of CFAP. Core‐level XPS spectra of k) Fe 2p, l) Co 2p, and m) Au 4f. n) Vis‐NIR absorption spectra of CFP, Au and CFAP. o) N_2_ adsorption–desorption isotherms of CFP. p) Pore size distribution of CFP corresponding to panel (o).

To develop CFAP, CoFe_2_O_4_ was first prepared through a standard solvothermal method.^[^ ^42^, ^43^ ^]^ FeCl_3_·6H_2_O and CoCl_2_·6H_2_O were used as the iron and cobalt sources, respectively. With the assistance of polyvinylpyrrolidone (PVP) as the capping agent, CoFe_2_O_4_ was catalytically synthesized in the presence of CH_3_ONa as alkaline source. On this basis, Au nanodots were in situ grown on the surface of porous CoFe_2_O_4_ by one‐step reducing HAuCl_4_ with NaBH_4_.^[^ ^44^ ^]^ Upon the formation of CoFe_2_O_4_@Au (CFA), surface PEGylation was further conducted to improve the biocompatibility and aqueous stability. As displayed by transmission electron microscopic (TEM) images, CFAP possesses well‐defined flower‐like structure with an average size of ≈75 nm (**Figure** **1**a,b). Nano‐sized tiny Au with a mean particle diameter of ≈4 nm is effectively embedded into the porous matrix of CoFe_2_O_4_ (Figure S1, Supporting Information). The size of Au nanodots is located in the effective range for acquiring optimal GOx‐mimic activities.^[^ ^45^, ^46^ ^]^ High‐resolution TEM images show the interplanar spacings of 0.21 and 0.23 nm in the lattice fringe, which are perfectly assigned to the inverse spinel phase plane (400) of CoFe_2_O_4_ and crystal face (111) of face‐centered cubic gold, respectively (Figure 1c). The electron diffraction pattern of CFAP also displays the polycrystalline concentric rings, which corresponds well with the crystal lattice of CoFe_2_O_4_ and Au (Figure 1d). Such regular particle‐like structure of CFAP with the decoration of Au nanodots is also revealed from scanning electron microscopy (SEM) (Figure 1e).

The hydrodynamic sizes of CoFe_2_O_4_, CFA and CFAP were measured to be 294, 294.4, and 324 nm, respectively, from dynamic light scattering (DLS) (Figure 1f; Figure S2, Supporting Information). Compared with the dehydrated particles, the slightly large hydrodynamic size of CFAP can be attributed to the influence of amplified Brownian motion that is coupled to hydrodynamic interaction at the solid‐liquid interface. To evaluate the stability of CFAP under aqueous conditions, CFAP was dispersed in deionized (DI) water, phosphate buffer saline (PBS) or Dulbecco's Modified Eagle Medium (DMEM, with 10% fetal bovine serum), followed by dynamically recording the hydrodynamic sizes from DLS during an incubation period of seven days. There is no obvious size fluctuation of CFAP over time, attesting to a steady state of CFAP in long‐term storage (Figure S3, Supporting Information). Zeta potentials of the intermediate and final products were documented to their colloidal stability (Figure 1g). Compared with the more negative charged CoFe_2_O_4_ (ζ potential: −10.62 mV), such parameter is significantly increased to −5.36 mV after Au immobilization. After surface PEGylation, ζ potential of CFAP is further elevated to −0.98 mV, which is related to the expose of positively charged amino residues on the particle surface. To decipher the crystallographic form of CFAP, its crystal structure and orientation were characterized by X‐ray powder diffraction (XRD) (Figure 1h). The crystalline features of CFAP match well with those of inverse spinel CoFe_2_O_4_ (JCPDS No.22‐1086) and face‐centered cubic Au (JCPDS No.04‐0784), which are in good accordance with the lattice fringe indexes in high‐resolution TEM images. Fourier‐transform infrared (FTIR) spectroscopy was carried out to verify the effective conjugation of PEG onto CFA (Figure 1i). Both CoFe_2_O_4_ and CFA exhibit intense vibration absorption peaks of Fe─O and Co─O at the wavenumber of 589.96 cm^−1^. By contrast, emerging absorption peaks at 1153.3 and 1217.3 cm^−1^ assigned to C─O stretching vibrations and one independent absorption peak at 2888.1 cm^−1^ belonging to C─H stretching vibrations are observed from the FTIR spectrum of CFAP, testifying to the successful surface PEGylation on CFA.

Elemental composition of CFAP was uncovered by analyzing the binding energies and different orbital peak locations from X‐ray photoelectron spectroscopy (XPS). The existences of elemental carbon (C), oxygen (O), iron (Fe), cobalt (Co), and gold (Au) are interpreted from the full‐survey XPS spectrum of CFAP (Figure 1j). Binding energies at 723.7 and 710.6 eV are assigned to the orbits of Fe 2p_1/2_ and Fe 2p_3/2_, respectively (Figure 1k).^[^ ^47^ ^]^ The specific satellite peaks of Fe(III) are centered at 734.28 and 717.98 eV. These data confirm the presence of trivalent Fe^3+^ in CFAP. Binding energies at 795.7 and 780.1 eV are assigned to the orbits of Co 2p_1/2_ and Co 2p_3/2_, respectively (Figure 1l).^[^ ^48^ ^]^ The specific satellite peaks of Co(II) are centered at 803.28 and 787.08 eV, indicating the presence of divalent Co^2+^. Two primary peaks located at 87.68 and 83.98 eV belong to the two orbits of Au 4f_5_ and Au 4f_7,_ confirming the existence of elemental Au (Figure 1m).^[^ ^49^ ^]^ Lastly, binding energy at 529.5 eV explicitly affirms the formation of metal oxide in CFAP (Figure S4, Supporting Information). The weight percent of Au in CFAP was measured to be 0.539% from inductively coupled plasma mass spectrometry (ICP‐MS). Raman spectroscopy was performed to further resolve the metal ligand bonds in CFA (Figure S5, Supporting Information). The strong absorption bands at Raman shift of 315, 470, 620, and 670 cm^−1^ corresponds well to cobalt ferrites. There are slight location shift and intensity change of these peaks in CFA, which could be interpreted as the influence of surface plasmon resonance effect from Au nanodots. The intense broad peak at 609 cm^−1^ in CFA is assigned to Au component. As for spectral characteristics, there are no representative absorption peaks in the vis‐NIR spectroscopic spectrum of CoFe_2_O_4_ (Figure 1n). By contrast, Au and CFAP produce representative absorption peaks at 520 and 570 nm, respectively. The position wavelength of absorbance peak is strongly dependent on the size of Au particles. Brunauer‐Emmett‐Teller (BET) theory was carried out to analyze the porosity and pore size distribution. The specific surface area of CFP was determined to be 64.24 m^2^ g^−1^ (Figure 1o). Additionally, the pore diameter of CFP was calculated to be 2.67 nm (Figure 1p). The mesoporous structure is tremendously favorable for the anchoring Au nanodots during the synthesis of CFAP.

### 2.2 Catalytic Activities and Underlying Mechanisms

**Figure 2:**
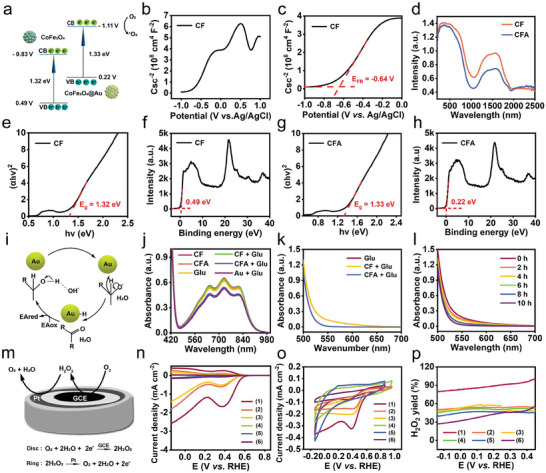
Energy band structure of CFA and evaluation of its GOx‐mimic activity. a) Energy band diagram of CoFe_2_O_4_ and CFA. b) Mott‐Schottky characteristic curve of CoFe_2_O_4_. c) Flat‐band potential derived from Mott‐Schottky measurement. d) UV–vis–NIR DRS of CF and CFA. e) Tauc plot of CoFe_2_O_4_ derived from the corresponding diffuse reflectance spectrum. f) VB‐XPS spectrum of CoFe_2_O_4_. g) Tauc plot of CFA derived from the corresponding diffuse reflectance spectrum. h) VB‐XPS spectrum of CFA. i) GOx‐mimic catalytic mechanism of CFA. j) Optical absorption spectrum of ABTS^+·^ containing Au, CoFe_2_O_4_ (200 µg mL^−1^) or CFA (200 µg mL^−1^) in the presence of glucose after reaction for 5 min. k) Optical absorption spectrum of DNS containing CF (200 µg mL^−1^) or CFA (200 µg mL^−1^) in the presence of glucose after reaction for 8 h. l) Optical absorption spectrum of DNS containing CFA (200 µg mL^−1^) in the presence of glucose after reaction for various periods (0–10 h). m) Schematic diagram to illustrate RRDE measurement for selective oxygen reduction through an electrochemical workstation. n) LSV plots by applying proper potential ranges at the scan rate of 10 mV s^−1^, measured by RRDE in saturated Na_2_SO_4_ solution (0.5 m). o) CV curves at the scan rate of 10 mV s^−1^ in terms of different groups. p) Relative H_2_O_2_ yield derived from the oxygen reduction data from panels (n) and (o). Groups are allocated to be (1) CFA + Glu + O_2_, (2) CFA + O_2_, (3) O_2_, (4) CFA + Glu + N_2_, (5) CFA + N_2_, (6) N_2_.

Modulation of the energy band structure of Schottky junction has a profound effect on its stimuli‐responsive properties. To this end, electronic band structure of CoFe_2_O_4_ and CFA was figured out by determining the potentials of valence and conduction band edge (**Figure** **2**a). The positive slope of Mott‐Schottky curve is indicative of N‐type semiconductor of CoFe_2_O_4_ and its flat‐band potential was measured to be −0.64 V (Figure 2b,c). To elucidate the charge carrier migration in semiconductor, energy level distribution of CoFe_2_O_4_ and CFA were measured by UV–vis–NIR diffuse reflectance spectroscopy (DRS) and valence band X‐ray photoelectron spectroscopy (VB‐XPS) (Figure 2d). First, Tauc plot of the modified Kubelka‐Munk (KM) function was depicted to determine the bandgap energy, which is quantified to be the x‐axis intercept of extrapolated linear part of curve. The energy bandgap of CoFe_2_O_4_ was measured to be 1.32 eV (Figure 2e). The valence band edge of CoFe_2_O_4_ was computed to be 0.49 V versus normal hydrogen electrode (NHE) by linearly extrapolating the leading edge to the extended base line of the VB‐XPS spectra (Figure 2f). Correspondingly, the conduction band edge of CoFe_2_O_4_ was estimated to be −0.83 V. Similarly, energy bandgap, valence band edge and conduction band edge of CFA were calculated to be 1.33 eV, 0.22 V, and −1.11 V, respectively (Figure 2g,h). Obviously, Au‐incorporation does not alter the bandgap energy of CoFe_2_O_4_ but only cause the change in Fermi level. The conduction band edge of CFA is more negative than the redox couple potential of O_2_/·O_2_^–^ (−0.33 V), which allows US‐triggered ·O_2_^–^ production and subsequent ^1^O_2_ generation. The narrow bandgap of CFA would benefit the rapid separation of e^−^/h^+^ pairs. Meanwhile, Au nanodots can serve as the electron scavengers to prevent the recombination of charge carriers and improve the electron utilization.

The reaction pathway of CFA is the same as that of nature GOx, except that a Brønsted base of OH^−^ is used to attract H^+^ from glucose (Figure 2i).^[^ ^50^ ^]^ To investigate the GOx‐mimic activity of CFA, ABTS^+·^ was used as both an electron acceptor and a colorimetric indicator for one‐step detection of glucose (Figure S6a, Supporting Information). In the presence of glucose, CFA could catalyze the reduction of ABTS^+·^, resulting in the absorbance decrease at 734 nm. Such deduction in the absorption intensity of ABTS^+·^ is negatively correlated with glucose concentration, having the merits of high linearity and low detection limit. “CF + Glu” results in an insignificant change in the optical absorption of ABTS^+·^, which is similar with the control groups without glucose addition (Figure 2j). By contrast, both “Au + Glu” and “CFA + Glu” lead to an obvious absorbance decrease of ABTS^+·^ at 734 nm, implying an effective glucose consumption in a short period of 5 min. Such glucose depletion is also found be related to the substrate concentration and reaction time (Figure S6b–d, Supporting Information). Alternatively, 3,5‐dinitrosalicylic acid (DNS) assay was employed for the estimation of glucose level after various treatments. The glucose amount can be reflected by the absorption intensity of DNS at 510 nm. Compared with “CF + Glu”, a considerable decrease in DNS absorbance is caused by “CFA + Glu”, indicating an effective exhaustion of glucose by CFA (Figure 2k). Such DNS absorbance decrease is also dependent on the glucose concentration and reaction time (Figure 2l; Figures S7 and S8, Supporting Information). All previous findings validate the GOx‐mimic activity of CFA, stemming from the intrinsic catalytic property of Au nanozyme.

To further decipher the CFA‐mediated glucose oxidation and coupled oxygen reduction reaction (ORR), a rotating ring disk electrode (RRDE) was used to elucidate the electron transfer process (Figure 2m). CFA was loaded on the glass carbon disk electrode (GCE) in the center and a potential of 1.2 V was applied on the Pt ring electrode. In a linear negative scan rate of 10 mV s^−1^, O_2_ would be reduced by 2e^−^ to produce H_2_O_2_. H_2_O_2_ can be captured by the Pt ring electrode and further oxidized to O_2_. Thus, the amount of H_2_O_2_ yield can be determined by the oxidation current at the ring electrode. Linear sweep voltammetry (LSV) and cyclic voltammetry (CV) plots reveal the highest H_2_O_2_ production from the treatment of “CFA + Glu + O_2_”, as indicated by the highest density of real‐time current (Figure 2n,o). The electron transfer number of CFA‐mediated redox reaction is estimated to be two, further evidencing the H_2_O_2_ generation from O_2_ reduction (Figure 2p; Figure S9, Supporting Information). Such high‐efficient glucose exhaustion by CFA shows good clinical promise for the starvation of cancer cells.

### 2.3 ROS Generation Capacity of CFA

**Figure 3:**
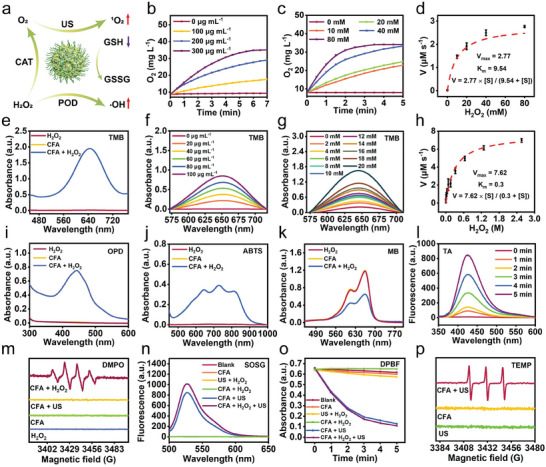
ROS generation properties of CFA. a) Schematic diagram to illustrate the enzymatic activities of CFA for inducing redox dyshomeostasis. b) Dissolved oxygen level in H_2_O_2_ solution (10 mm) containing CFA at different concentrations (0–300 µg mL^−1^) over time. c) Dissolved oxygen level in H_2_O_2_ solution at different concentrations (0–80 mm) containing CFA over time. d) Fitting curve of CAT‐mimic enzymatic kinetics of CFA derived from Michaelis‐Menten equation. e) Optical absorption spectra of TMB (125 µg mL^−1^) containing CFA (200 µg mL^−1^) or/and H_2_O_2_ (10 mm) after reaction for 10 min. f) Optical absorption spectra of TMB (125 µg mL^−1^) containing CFA at different concentrations in the presence of H_2_O_2_ (10 mm) after incubation for 10 min. g) Optical absorption spectra of TMB (125 µg mL^−1^) containing CFA (200 µg mL^−1^) in the presence of H_2_O_2_ at different dosages (0–20 mm) after reaction for 3 min. h) Fitting curve of POD‐mimic enzymatic kinetics of CFA derived from Michaelis‐Menten equation. Optical absorption spectra of i) OPD (25 µg mL^−1^) and j) ABTS (25 µg mL^−1^) containing CFA (50 µg mL^−1^) or/and H_2_O_2_ (10 mm) after reaction for 10 min. k) Optical absorption spectra of MB (10 µg mL^−1^) containing CFA (200 µg mL^−1^) or/and H_2_O_2_ (10 mm) after reaction for 1 h. l) Fluorescence emission spectra of TA (12.5 µg mL^−1^) containing CFA (100 µg mL^−1^) in the presence of H_2_O_2_ (10 mm) after reaction for various periods (0–5 min, λ_ex_: 315 nm). m) ESR spectra to confirm the yield of ·OH in the presence of DMPO (25 mg mL^−1^) as the radical trapping agent after various treatments. n) Fluorescence spectra of SOSG (5 µm) containing CFA (600 µg mL^−1^) and/or H_2_O_2_ (10 mm) after US irradiation for (1.0 MHz, 1.0 W cm^−2^, 50% duty cycle) for 5 min (λ_ex_ = 488 nm). o) Absorbance decay of DPBF (10 µg mL^−1^) at 417 nm in the presence of CFA (200 µg mL^−1^) and/or H_2_O_2_ (10 mm) after US irradiation for (1.0 MHz, 1.0 W cm^−2^, 50% duty cycle) for up to 5 min. p) ESR spectra to confirm the yield of ^1^O_2_ in the presence of TEMP (400 mm) as the radical trapping agent after diverse treatments.

Taking advantages of the splendid energy bandgap structure and the presence of bimetallic Fe/Co with Fenton properties, CFA is expected to possess multi‐catalytic properties to aggravate redox dyshomeostasis (**Figure** **3**a). In consideration of the good electronic conductivity and large active surface area, the catalase (CAT)‐mimic activity of CFA was first investigated by measuring dissolved O_2_ level in H_2_O_2_ solution. An instant O_2_ generation is observed during the first 4 min and the dissolved O_2_ level reaches equilibrium in the following 3 min (Figure 3b). Moreover, the oxygen yield rate is strongly dependent on the concentrations of CFA and H_2_O_2_ (Figure 3c). To numerically investigate the CAT‐like activity of CFA toward H_2_O_2_ decomposition, Michaelis–Menten saturation curve was plotted to depict the steady‐state kinetics (Figure 3d; Figure S10, Supporting Information). V_max_ signifies the maximum reaction velocity when the enzyme is saturated with its substrate, and the Michaelis constant (K_M_) represents the concentration of substrate that provides 50% of V_max_. The V_max_, K_M_ and catalytic efficiency describing the CAT‐mimic activity of CFA were calculated to be 2.77 µm s^−1^, 9.54 mm, and 0.687 m (M s)^−1^, respectively (**Table** **1**). The admirable catalase‐mimic activity of CFA may contribute to reverse the hypoxic TME and profoundly increase the ^1^O_2_ yield during sonosensitization.

**Table 1:** Kinetic parameters for enzyme‐mimicking activity of CFA.

	V_max_ [µm s^−1^]	K_m_ [mm]	K_cat_ [s^−1^]	Efficiency [m/(M·s)]
CAT	2.77	9.54	6.55 × 10^−4^	0.687
POD	7.62	0.30	9.02 × 10^−4^	30.1

In the light of the enhanced Fenton‐like reaction mediated by Fe/Co metallic pairs, peroxidase (POD)‐mimic activity of CFA was further validated via multiple colorimetric methods. In the presence of H_2_O_2_, CFA leads to an outstanding increase in the oxidized 3,3′,5,5′‐tetramethylbenzidine (oxTMB) absorbance at 652 nm, manifesting a massive ·OH production (Figure 3e; Figure S11, Supporting Information). In addition, the yield rate of ·OH is strongly related to the concentrations of CFA and H_2_O_2_, as well as the reaction time (Figure 3f,g). Such POD‐mimic activity of CFA was subsequently numerically investigated by determining the steady‐state enzyme kinetics. Based on the Michaelis‐Menten saturation curve, V_max_, K_M,_ and catalytic efficiency describing its POD‐mimic activity were calculated to be 7.62 µm s^−1^, 0.30 mm, and 30.1 m (M s)^−1^, respectively (Figure 3h and Table 1). On this basis, ·OH generation by CFA was further investigated through colorimetric assays of o‐phenylenediamine (OPD), 2,2′‐azino‐di‐(3‐ethylbenzthiazoline sulfonic acid) (ABTS), methylene blue (MB) and terephthalic acid (TA) (Figure 3i–l). The fantastic POD‐mimic activity of CFA is evidenced by the intense absorbance increase at 435 nm for OPD and 734 nm for ABTS, the significant absorbance reduction at 665 nm for MB, as well as the strong fluorescence emission for TA (λ_ex_/λ_em_ = 315/426 nm) (Figure S12, Supporting Information). To identify the specific ·OH generation from CFA‐mediated Fenton‐reaction, electron spin spectroscopy (ESR) was implemented for ·OH detection upon the addition of 5, 5‐dimethyl‐1‐pyrroline N‐oxide (DMPO) (Figure 3m). In the presence of H_2_O_2_, CFA can effectively render the production of ·OH, as verified by the intense ESR signal with amplitude ratio of 1:2:2:1 (Figure 3m). Compared with the unitary Fe or Co elements, the heterostructure of bimetallic oxides (Fe/Co) can accelerate charge transfer from colloidal interface to Fenton catalysts and increase the electron concentration in Fenton reaction center, which may promote H_2_O_2_ conversion to ·OH.

Provided by the redox reaction by the hot electrons at the conduction band edge of CFA, US‐triggered ^1^O_2_ generation was thereby studied via manifold colorimetric methods. Singlet oxygen sensor green (SOSG) fluorescent probe was first used to specifically detect the production of ^1^O_2_. “CFA + US” contributed to a striking ^1^O_2_ yield, as evidenced by the distinctly high fluorescence emission at 525 nm (Figure 3n). Furthermore, the addition of H_2_O_2_ can further augment the ^1^O_2_ production, owing to the local oxygenation in ambient environment. The amount of yielded ^1^O_2_ is closely related to the concentrations CFA and H_2_O_2_, as well as the reaction time (Figure S13, Supporting Information). Alternatively, 1,3‐diphenylisobenzofuran (DPBF) as another ^1^O_2_ trapping‐agent was used for singlet oxygen sensing. Similar with the SOSG assay, the largest quantify of ^1^O_2_ molecules is generated by “CFA + US + H_2_O_2_”, as evidenced by the most significant absorbance decay of DPBF at 417 nm (Figure 3o). To identify of ROS category, ESR was carried out to detect the short‐lived ^1^O_2_ in the presence of 2, 2, 6, 6‐tetramethyl‐4‐piperidine (TEMP). As expected, “CFA + US” produces a distinct characteristic signal with an amplitude ratio of 1:1:1, indicating the formation TEMP/^1^O_2_ adducts (Figure 3p, Supporting Information). The optimal charge transfer kinetics of interfacial redox reaction for ^1^O_2_ generation can benefit from enhanced separation and retarded recombination of e^−^/h^+^ pairs during sonosensitization. It is also proved that “CFA + US” also gives rise to the formation of other forms of ROS (e.g., superoxide anions) to exacerbate oxidative stress, as proved by the nitroblue tetrazolium (NBT) colorimetric assay (Figure S14, Supporting Information).

To explore whether GSH can be consumed by CFA to deteriorate ROS imbalance, the chromogenic substrate 5,5′‐dithiobis‐(2‐nitrobenzoic acid) (DTNB, Ellman's reagent) was used for GSH detection in virtue of the production of 2‐nitro‐5‐thiobenzoic acid (TNB) with bright yellow color. Based on Ellman's assay, CFA is demonstrated to effectively deplete GSH, and the consumption rates are closely correlated with the CFA concentration and reaction time (Figure S15, Supporting Information). The redox valence changes of the ion couples of Fe^2+/3+^ and Co^2+/3+^ could endow CPA with such GPx‐like activity. Not only promote redox dyshomeostasis for triggering apoptosis, unitary GSH exhaustion can also facilitate the accumulation of lipid peroxides to induce ferroptosis.

### 2.4 Cellular Uptake In Vitro

**Figure 4:**
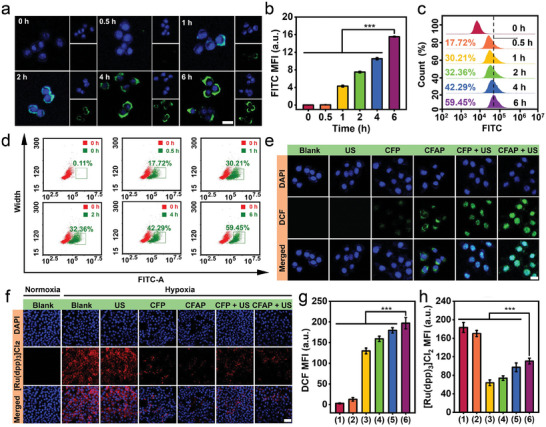
Cellular uptake in vitro. a) Confocal microscopy of 4T1 cells after incubation with FITC‐labeled CFAP for various periods (scale bar: 10 µm). b) Quantitative analysis of intracellular CFAP localization by analyzing the fluorescence intensity of FITC corresponding to panel (a). c) Flow cytometry histogram to exhibit the time‐dependent cellular uptake of CFAP. d) Flow cytometry dot plots corresponding to panel (c). e) Confocal microscopy of 4T1 cells after various treatments and staining with DCFH‐DA probe for intracellular ROS detection (scale bar: 10 µm). f) Confocal microscopy of 4T1 cells after diverse administrations in hypoxia or normoxia, followed by staining with [Ru(dpp)_3_]Cl_2_ oxygen sensing probe (scale bar: 40 µm). g) Quantitative analysis of DCF fluorescence corresponding to panel (e). h) Quantitative analysis of [Ru(dpp)_3_]Cl_2_ fluorescence corresponding to panel (f). Groups are allocated to be (1) Blank, (2) US, (3) CFP, (4) CFAP, (5) CFP + US, (6) CFAP + US. Data are displayed as mean ± SD (n = 4). ^***^ *p* < 0.001.

Efficient cellular uptake is a prerequisite for nanoparticles fulfilling their therapeutic functions for tumor inhibition. To this end, endocytosis behavior of CFAP by 4T1 murine mammary cancer cells was investigated in multiple perspectives. To study its intracellular distribution, CFAP was first covalently labeled with fluorescein isothiocyanate (FITC) as a fluorescence tracker (Figure S16, Supporting Information). Based on confocal microscopy, fluorescence of CFAP in cytosolic region is progressively enhanced with the incubation time, suggesting that a high period dependency of endocytosis (**Figure** **4**a,b). Furthermore, cellular uptake of CFAP was quantitatively analyzed by flow cytometry (Figure 4c). The percentage of 4T1 cells that take in a significant number of CFAP reaches up to 59.45% after 6 h incubation, indicating a rapid cellular internalization of nanoparticles to perform therapeutic effect (Figure 4d). To investigate the capability of CFAP in causing oxidative stress, 2′‐7′‐dichlorofluorescein diacetate (DCFH‐DA) fluorescent probe was used to detect the cytosolic ROS level (Figure 4e). In the presence of ROS, DCFH‐DA as a cell‐permeant tracer can be instantly oxidized to 2′,7′‐dichlorofluorescein (DCF) with vivid green fluorescence. In contrast to the untreated group, the administration of CFAP effectively increases intracellular ROS amount, as evidenced by the prominent DCF fluorescence emission, which is associated with the ·OH yield from Fe/Co‐mediated Fenton‐like reactions. In comparison, US irradiation further amplifies the cytosolic ROS content under the treatment of CFA or CFAP, attributed to the additional ^1^O_2_ generation from sonosensitization. Thanks to the optimum energy band structure of CFAP, “CFAP + US” contributes to the distinctly high ROS yield in cytoplasmic region, manifesting the massive production of ROS from the sonodynamic and chemodynamic processes.

Hypoxia in TME resulting from abnormal vascular network and altered tumor metabolism gives rise to angiogenesis and invasiveness, eventually promoting metastasis, which strikingly reduces O_2_‐consuming therapeutic effectiveness and exacerbates drug resistance. To evaluate the capability of CFAP in hypoxia reversal at the cellular level, [Ru(dpp)_3_]Cl_2_ oxygen sensing probe was exploited to probe the oxygenation status based on fluoresce microscopy (Figure 4f). Strong red fluorescence is observed from cytosolic region in oxygen‐deficient environment, which verifies the availability of [Ru(dpp)_3_]Cl_2_ for hypoxia identification. Distinctly, such red fluorescence is significantly quenched in 4T1 cells with the treatment of CFA or CFAP, indicating that the oxygenation level is restored to normoxia, which is attributed to the H_2_O_2_ decomposition by CoFe_2_O_4_ with intrinsic catalase‐mimic activity. It is worth to note that US irradiation slightly decreases the intracellular oxygenation level on the condition of CFAP administration, originating from the O_2_ consumption during sonosensitization. The results of DCFH‐DA assay and [Ru(dpp)_3_]Cl_2_‐based oxygenation probing are also semi‐quantitatively interpreted through determining their respective mean fluorescence intensity (MFI) in vitro (Figure 4g,h).

### 2.5 Cytotoxicity of CFAP In Vitro

**Figure 5:**
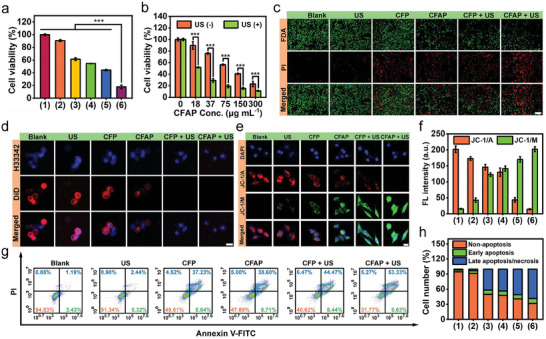
Cytotoxicity of CFAP in vitro. a) Viability of 4T1 cells after various administrations for 24 h. b) Viability of 4T1 cells after treatment with CFAP at different concentrations for 6 h, followed by US irradiation (1.0 MHz, 0.5 W cm^−2^, 20% duty cycle) for 5 min where applicable. c) Fluorescence microscopy of 4T1 cells after different treatments, followed by co‐staining with FDA/PI. Live and dead cells are represented by green and red dots, respectively (scale bar: 200 µm). d) Confocal microscopy of 4T1 cells after receiving diverse regimens and staining with DiD membrane dye (scale bar: 10 µm). e) Confocal microscopy of 4T1 cells after manifold treatments and staining with JC‐1 (scale bar: 10 µm). f) Fluorescence intensity of JC‐1/M and JC‐1/A corresponding to panel (e). g) Flow cytometry dot plots to investigate the cell apoptotic status in terms of different groups via Annexin V‐FITC Apoptosis Detection Kit. Annexin V‐FITC(‐)/PI(‐): non‐apoptotic cells; Annexin V‐FITC(+)/PI(‐): early apoptotic cells; Annexin V‐FITC(+)/PI(+): late apoptotic cells; Annexin V‐FITC(‐)/PI(+): necrotic cells or cell debris. h) Histogram to quantify the number of cells in non‐apoptosis, early apoptosis, late apoptosis and necrosis stages corresponding to panel (g). Groups are allocated to be (1) Blank, (2) US, (3) CFP, (4) CFAP, (5) CFP + US, (6) CFAP + US. Data are displayed as mean ± SD (*n* = 4). ^***^ *p* < 0.001.

Quantified biocompatibility of nanoparticles on healthy cells is essential for their being applied in clinical trials and eventual medical utilizations. Given all this, cytotoxicity of CFAP toward normal somatic cells, including human umbilical vein endothelial cells (HUVECs) and L929 murine fibroblasts (L929s), was investigated via standard 3‐(4,5‐dimethylthiazol‐2‐yl)−2,5‐diphenyltetrazolium bromide (MTT) assay (Figure S17, Supporting Information). After 24 h incubation, viabilities of HUVECs and L929s were measured to be more than 85%, even at a considerably high CFAP concentration of 300 µg mL^−1^, testifying to the decent biocompatibility of CFAP. Based on this, tumor‐specific cytotoxicity of CFAP was investigated on murine mammary carcinoma 4T1 cells. Obviously, the treatment of CFP or CFAP causes a certain degree of cell death, which is associated with the oxidative damage from ·OH accumulation during Fe/Co‐mediated Fenton reaction (**Figure** **5**a). Compared with CFP, a slightly lower cell viability is achieved by CFAP administration, resulting from the massive H_2_O_2_ supply from intracellular glucose oxidation. When applying US irradiation, the most prominent cell death is obtained with the mediation of CFAP, as reflected by the extremely low cell viability of 18.45%, which manifests the efficacious combinatorial CDT and SDT effects. Such CFAP‐induced cytotoxicity against tumor cells also shows a dosage‐dependent manner (Figure 5b). Taking account of GSH‐depleting capability of CFAP, its inducibility on ferroptosis was explored by using a ferroptosis‐selective inhibitor of ferrostatin‐1 (Fer‐1). Clearly, the addition of Fer‐1 rescues 4T1 cells from the damage by “FPR + US”, substantiating the occurrence of ferroptosis during CFAP‐mediated treatment (Figure S18, Supporting Information).

To directly visualize the viable status of 4T1 cells after various treatments, live/dead cells were co‐stained with fluorescein diacetate (FDA) and propidium iodide (PI) for fluorescence identification (Figure 5c). Distinctly, the almost undetectable green fluorescence and the most intense red fluorescence are discovered in the group of “CFAP + US”, verifying the most severe morbidity of 4T1 cells (Figure S19, Supporting Information). To reveal the structure integrity of cell membrane, an NIR fluorescence probe of DiD was used for specifically staining (Figure 5d). Noticeably, “CFAP + US” results in almost entire disintegration of plasma membrane, as evidenced by the undetectable red fluorescence of DiD probe in cytosolic region (Figure S20, Supporting Information). The activation of innate apoptotic pathways always stems from mitochondrial pathogenesis. To this end, mitochondrial dysfunction was characterized by monitoring the change of mitochondrial membrane potential (MMP), with the aid of JC‐1 assay kit (Figure 5e). JC‐1 displays MMP‐dependent enrichment in mitochondria, where dye molecules form JC‐1 aggregates (JC‐1/A, λ_ex_/λ_em_ = 435/590 nm) in normal mitochondria and retain to be JC‐1 monomers (JC‐1/M, λ_ex_/λ_em_ = 485/527 nm) in damaged mitochondria. Distinctly, “CFAP + US” leads to marvelous mitochondrial dysfunction upon observing the most significant red fluorescence reduction and green fluorescence intensification (Figure 5f; Figure S21, Supporting Information). On this basis, Annexin V‐FITC/PI staining was implemented to uncover the apoptotic level of 4T1 cells, arising from mitochondrial damage (Figure 5g). Flow cytometry results indicate that the maximum percentage of apoptosis/necrosis at 68.23% is realized by “CFAP + US”, in virtue of the most deleterious mitochondrial disorder and plasma membrane injury (Figure 5h). Alternatively, the expression of cytosolic cleaved caspase‐3 (a central enzyme in apoptosis) after diversiform treatments was determined from Western blot (WB) analysis (Figure S22, Supporting Information). As expected, the highest level of cleaved caspase‐3 expression is achieved by “CFAP + US”, originating from the most severe mitochondrial damage under the intense redox dyshomeostasis. All these results perfectly match with the findings in MTT assay, implying the incredible potency of CFAP for high‐performance tumor‐specific cell destruction.

### 2.6 DAMPs Release In Vitro

**Figure 6:**
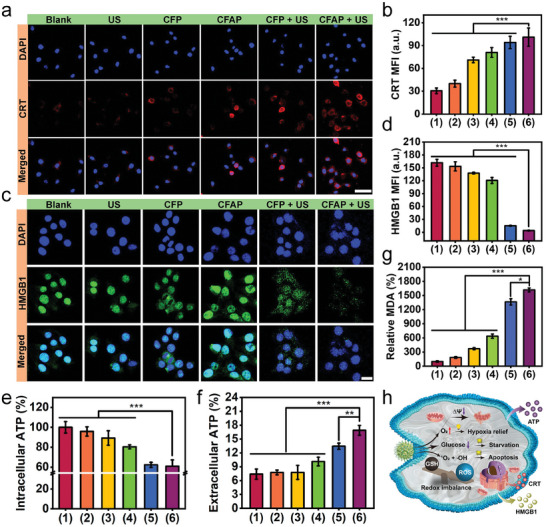
DAMPs release in vitro. a) Confocal microscopy of 4T1 cells after various treatments and immunostaining with CRT (scale bar: 20 µm). b) Quantitative analysis of CRT fluorescence corresponding to panel (a). c) Confocal microscopic images of 4T1 cells after diverse administrations and immunostaining with HMGB1 (scale bar: 10 µm). d) Quantitative analysis of HMGB1 fluorescence corresponding to panel (b). The levels of e) intracellular ATP and f) extracellular ATP in terms of different treatment groups, measured by ATP assay kit. g) Relative intracellular MDA level after diverse treatments. h) Scheme to illustrate the DAMPs release from 4T1 cells during CFAP‐mediated therapy. Groups are allocated to be (1) Blank, (2) US, (3) CFP, (4) CFAP, (5) CFP + US, (6) CFAP + US. Data are displayed as mean ± SD (*n* = 4). ^*^ *p* < 0.05, ^**^ *p* < 0.01, ^***^ *p* < 0.001.

ICD has gained increasing visibility for how it impacts the innate and adaptive immune responses. The distinct characteristic of ICD is the release of damage associated molecular patterns (DAMPs) owing to the plasma membrane damage or complete rupture, which significantly mediate the progression and magnitude of this process. To evaluate the DAMPs release from tumor cells during CFAP‐mediated therapy, an optimized protocol of immunostaining was implemented after cells receiving various treatments (Figure S23, Supporting Information). During ICD, calreticulin (CRT) as an endoplasmic reticulum (ER)‐resident recognition ligand can be translocated onto cytomembrane, which further binds and activate LDL‐receptor‐related protein (LRP) on phagocytes. Confocal microscopy indicates the most CRT translocation caused by “CFAP + US”, as reflected by the most intense red fluorescence on the membrane of 4T1 cells (**Figure** **6**a,b). These findings prove that CFAP administration can effectively trigger ecto‐CRT translocation through pronounced ER‐stress. In another aspect, the nucleus‐to‐cytoplasm migration of high mobility group box protein 1 (HMGB1) in damaged cells can promote the maturation of dendritic cells and stimulate the secretion of inflammatory factors to elicit proinflammatory responses. Opposing to the native residence of HMGB1 in the nucleic region of untreated cells, an arresting leakage of HMGB1 to cytoplasm and extracellular milieu is caused by “CFAP + US”, as evidenced by the drastic attenuation of green fluorescence in cytosolic region (Figure 6c,d). ICD also initiates the release of adenosine triphosphate (ATP) and purinergic signaling in NLRP3 inflammasome activation. To this end, the amounts of intracellular and extracellular ATP after various treatments were measured through ATP assay kit (Figure 6e,f). As noted, “CFAP + US” results in the marvelous release from cytosolic region, which may be ascribed to the irreversible damage of plasma membrane (Figure S24, Supporting Information). GSH depletion always causes the downregulation of GPx4, which may induce massive lipid peroxidation and increase free radical‐associated damage to membrane structures. In the light of this, Western blot analysis confirms a moderate decrease of cytosolic GPx4 level by the treatment of CFAP, and such effective downregulation of GPx4 expression can be associated with the deficiency of endogenous GSH (Figure S22, Supporting Information). The disorder of GPx4 may impair the removal of excess lipid peroxides, which would exert toxicity through propagating ROS generation and directing DNA/protein damage. Lipid peroxidation is also regarded as a hallmark of ferroptosis and is strongly associated with the damage of cytomembranes. In this regard, the accumulation of lipid peroxides in cytosol was assessed by MDA assay kit. As expected, the highest level of lipid peroxidation is provoked by “CFAP + US”, as evidenced by the largest amount of MDA enrichment (Figure 6g; Figure S25, Supporting Information). All above results demonstrate the effective induction of DAMPs release by CFAP administration, and the proved ICD is imperative for stimulating antitumor immunity for interventional immunotherapy (Figure 6h).

### 2.7 Anti‐Metastasis In Vitro

**Figure 7:**
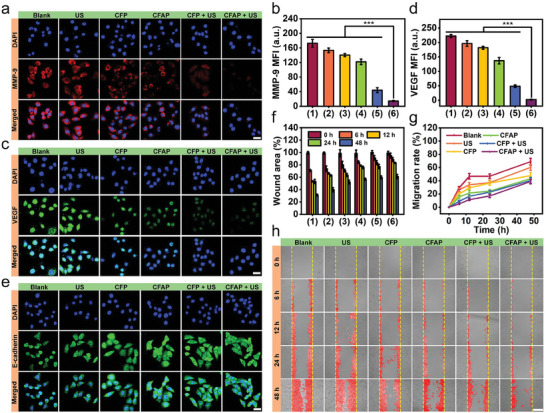
Anti‐metastasis evaluation in vitro. a) Confocal microscopy of 4T1 cells after various treatments and staining with MMP‐9 (scale bar: 10 µm). b) Quantitative analysis of MMP‐9 fluorescence corresponding to panel (a). c) Confocal microscopy of 4T1 cells after different administrations and staining with VEGF (scale bar: 10 µm). d) Quantitative analysis of VEGF fluorescence corresponding to panel (b). e) Confocal microscopy of 4T1 cells after diverse regimens and staining with E‐cadherin (scale bar: 10 µm). f) The percentage of wound area after various incubation periods in terms of different groups. g) Migration rates of 4T1 cells quantified from scratch assay. h) Bright field microscopic images of 4T1 cells at multiple time points succumbing to various treatments (scale bar: 200 µm). Groups are allocated to be (1) Blank, (2) US, (3) CFP, (4) CFAP, (5) CFP + US, (6) CFAP + US. Data are displayed as mean ± SD (*n* = 4). ^***^ *p* < 0.001.

Metastatic malignant neoplasm is always the major cause of therapy failure, which is featured as tumor migration and invasion into lymphatic or blood vessels, extravasation and colonization at distant sites. To explore the potential of CFAP in anti‐metastasis, the expression of metastasis‐related proteins, including matrix metalloproteinase‐9 (MMP‐9), vascular endothelial growth factor (VEGF) and cell‐adhesion molecule (E‐cadherin), were measured through immunofluorescence staining. MMP‐9 as a family of zinc‐dependent proteases can regulate the remodeling and degradation of various proteins in ECM, and promote the secretion of pro‐angiogenic factors for metastatic angiogenesis. VEGF is essential to promote angiogenesis based on enhancing the proliferation/survival and migration/invasion of endothelial cells, increasing permeability of existing blood vessels as well as forming a lattice metastatic network. E‐cadherin is a key component of the adherence junctions that is important for maintaining epithelial tissue integrity. A decreased level of E‐cadherin can promote cell migration and follow‐up metastasis. Distinctly, the most outstanding downregulation of MMP‐9 and VEGF is concomitant with the highest upregulation of E‐cadherin after the treatment of “CFAP + US”, manifesting the most effective blockade of molecular signaling pathways for metastasis (**Figure** **7**a–e). On this basis, the migration capacity of neoplasm was studied by implementing scratch‐wound healing assay on the confluent monolayer of 4T1 cells. Obviously, “CFAP + US” leads to the most significant restraint on the wound closure during the observation period of 48 h, which gives promise of an effectual inhibition on 2D cell migration (Figure 7f–h).

### 2.8 Tumor Inhibition In Vivo

**Figure 8:**
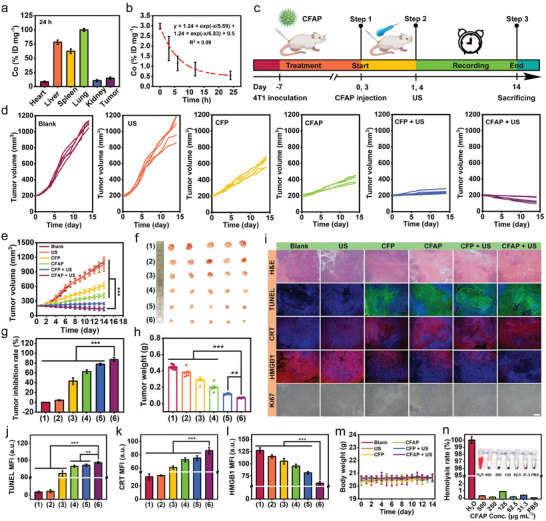
Tumor inhibition in vivo. a) Biodistribution of CFAP at 24 h post‐injection by quantifying the Co content via ICP‐MS. b) Blood level of CFAP over 24 h measured by ICP‐MS. c) Timing chart of the treatment procedures for animal experiments. d) Tumor growth curve by dynamically recording the tumor volume during the therapeutic course. e) Tumor volume change in terms of different groups. f) Digital photograph of the excised tumor on day 14. g) Tumor inhibition rates by the end of treatment. h) Tumor weight in terms of different groups on day 14. i) Pathological analysis of the excised tumor harvested on day 14 through staining histological tissue sections with H&E, Ki67, TUNEL, HMGB1, and CRT (scale bar: 100 µm). Quantitative analysis of the fluorescence intensities of j) TUNEL, k) CRT, and l) HMGB1 corresponding to panel (i). m) Mouse body weight during the treatment course of two weeks. n) Hemolysis rate of erythrocytes after incubation with CFAP at gradient concentrations for 6 h. Groups are allocated to be (1) Saline, (2) US, (3) CFP, (4) CFAP, (5) CFP + US, (6) CFAP + US. Data are displayed as mean ± SD (*n* = 5). ^**^ *p* < 0.01, ^***^ *p* < 0.001.

In the consideration of the desirable particle size of CFAP for enhanced permeation & retention (EPR) taking effect, intravenous injection was selected as the dosing approach for the treatment of animal models. Beforehand, biodistribution of CFAP was characterized to assess the tumorous enrichment by analyzing the content of Co through ICP‐MS. At 24 h postinjection, a prominent accumulation of CFAP in tumor is achieved at 14.59% ID mg^−1^ (**Figure** **8**a). Furthermore, drug pharmacokinetics was assessed by quantifying the plasma Co level, and a two‐compartment model was used to simulate the kinetics of CFAP clearance (Figure 8b). The half‐lives for drug absorption (distribution) and desorption (elimination) are quantified to be 5.59 and 6.83 h, respectively.

Inspired by the fantastic tumor killing performance of CFAP in vitro and its decent biodistribution in vivo, its therapeutic efficacy toward solid tumor was investigated on BALB/c mice bearing 4T1 tumors. The detailed treatment procedure is displayed as a time chart in Figure 8c. As the tumor volume reached ≈200 mm^3^, all the mice were randomly allocated into the following six groups, including 1) Saline, 2) US, 3) CFP, 4) CFAP, 5) CFP + US, and 6) CFAP + US. All the agents (25 mg kg^−1^, in saline) were intravenously injected into the tail vein of mice on day 0 and 3. US irradiation (1.0 MHz, 1.0 W cm^−2^, 50% duty cycle) was carried out against the tumor region for 5 min on day 1 and 4. The treatment duration was predesigned to be two weeks, during which tumor volume and mouse weight were dynamically recorded. Compared with the saline, CFAP causes a moderate tumor inhibition effect, resulting from CDT outcome from Fe/Co‐mediated Fenton‐like reaction (Figure 8d). In comparison, more significant therapeutic effects are achieved by “CFP + US” and “CFAP + US”, arising from the high‐grade apoptosis/ferroptosis induced by US‐triggered redox dyshomeostasis. Distinctly, the maximum tumor growth inhibition (TGI) index of 87.35% is attained by “CFAP + US” on day 14, implying that Au‐decoration on CoFe_2_O_4_ can effectively amplify the redox dyshomeostasis for rendering a promoted therapeutic efficacy (Figure 8e–g). Such tumor growth trend is also affirmed by digital photograph and weight measurement of the excised tumors on day 14 (Figure 8h).

Pathological analysis of the damage status of tumor tissues was performed through a variety of histological staining methods on day 14 (Figure 8i). Hematoxylin and eosin (H&E) staining testifies to the most severe chromatin condensation, nucleic fragmentation and dissolution upon the treatment of “CFAP + US”. Concurrently, minimal tumor proliferation index is uncovered by Ki67 immunohistochemistry staining, as indicated by the massive reduction of Ki67‐positive cells. In addition, the most outstanding apoptosis signaling is discovered in the same group through terminal deoxynucleotidyl transferase dUTP nick end labeling (TUNEL) staining, as revealed by the most intense DNA fragmentation, which is reflected by the pervasive green fluorescence emission. On the other hand, immunofluorescence staining of CRT and HMGB1 substantiates the most striking translocation of ecto‐CRT onto plasma membrane and HMGB1 secretion into extracellular milieu by the administration of “CFAP + US” (Figure 8j–l). Such efficient DAMPs emission indicates the occurrence of severe ICD, which can sensitize carcinoma with high immunogenicity for the follow‐up immunotherapy.

Qualified biosafety of nanodrugs is the first prerequisite prior to clinical transformations. Given this perspective, CFAP reliability for intravenous administration was explored in terms of multiple perspectives. During the treatment course, there is no dramatic change in the mouse body weight, implying a negligible effect of CFAP on the metabolism of experiment animals (Figure 8m). By the end of treatment, pathological characteristics of major organs (heart, liver, spleen, lung and kidney) was investigated through H&E staining (Figure S26, Supporting Information). No arresting histological abnormalities are observed in all groups, attesting a relatively minimal systemic damages on heathy tissue. Furthermore, complete blood count was implemented to measure the physiological and biochemical parameters after intravenous injection of CFAP into BALB/c mice. Major blood parameters are all located in the normal reference range on day 1, 3, 5, 7, and 14, suggesting the minimum rise of CFAP in causing chronic disorders (Figure S27, Supporting Information). Blood compatibility was investigated by the co‐incubation of erythrocytes with CFAP at different concentrations (0‐500 µg mL^−1^) for 6 h (Figure 8n). Thereafter, hemolysis rates of erythrocytes are calculated to be less than 1% even at a relatively high CFAP concentration of 500 µg mL^−1^, testifying to an admirable hemocompatibility of CFAP when contacting with blood. All these data demonstrate the pronounced biosafety of CFAP for the upcoming clinical utilizations for oncotherapy.

### 2.9 Immune Stimulation and Anti‐Metastasis In Vivo

Dendritic cells (DCs) are pivotal antigen‐presenting cells that play an important role in regulating the balance between immune activation and tolerance to tumor antigens. Taking account of the essential role of DCs in priming CD8^+^ T lymphocytes during tumor immunotherapy, the quantity of matured DCs in spleen was characterized through immunofluorescence staining (Figure S28a–c, Supporting Information). The largest amount of CD80^+^CD86^+^ DCs is observed in spleen sections under the treatment of “CFAP + US”, implying the highest potency for antigen cross‐presentation. CD8^+^ T lymphocytes are primary immune cells for systematically inhibiting tumor proliferation and disrupting metastasis by recognizing their unique surface antigens. The maximum number of CD8^+^ T lymphocytes is identified in both spleen and tumor sections with the administration of “CFAP + US”, implying an effective elicitation of antitumor immunity (Figure S28d–g, Supporting Information). All these findings testify to the capacity of CFAP in rejuvenating immune response, aiming at trafficking effecter T cells to distant sites and induce the abscopal effects for metastasis suppression.

Taking account of the high efficacy in stimulating systemic antitumor immunity during CFAP administration, its therapeutic performance in suppressing distant metastasis was thereby investigated on 4T1 tumor‐bearing BALB/c mice. As 4T1 cells are capable of metastasis to lung, liver and brain, the hepatic metastasis was thereby established by intravenous infusion of 4T1 cells into mouse models on day 8 after the medication of primary tumor (Figure S29a, Supporting Information). All the mice were euthanized to collect liver tissue on day 21. There are many anomalous, enlarged, and ulcerated metastatic nodules in saline group from visualizing digital photos, indicating representative detrimental malignancy (Figure S29b, Supporting Information). In contrast to the control groups, the tumorigenic nodules are scarcely observed in the liver tissue after the treatment of “CFAP + US”, manifesting the most effective prevention of metastasis (Figure S29c, Supporting Information). The prominent hepatic metastasis suppression by CFAP is also confirmed by histopathological analysis. H&E staining exhibits many microscopic metastatic nodules in liver sections from the saline group, which are featured with dense nuclear chromatin accompanied with atypia (Figure S29d, Supporting Information). On the contrary, only benign nodules with fine chromatin and well‐defined nuclear contours are found in the group of “CFAP + US”. On this basis, the expression levels of Oct4 (a prognostic marker of hepatic metastasis) in liver tissue were characterized by immunofluorescence staining (Figure S29e, Supporting Information). As expected, “CFAP + US” leads to the lowest level of Oct4 expression, as evidenced by the weakest red fluorescence in liver sections (Figure S29f, Supporting Information). Such robust inhibition on liver metastasis by CFAP could significantly profit from the reversal of immunosuppression from ICD.

## 3 Conclusion

In summary, CFAP is successfully developed as a versatile TME modulators for reinforced redox dyshomeostasis. CFAP occupies CAT‐, POD‐, and GPx‐mimic activities, which are devoted to hypoxia alleviation, ·OH production and GSH depletion. Benefiting from the desirable energy band structure, CFAP acts as a qualified sonosensitizer for ^1^O_2_ generation under US actuation. The decorated Au nanodots can effectively deplete glucose to replenish H_2_O_2_, which not only causes tumor starvation but provides ingredients for CDT and enhances oxygenation as well. Furthermore, Au nanodots also serve as the electron scavengers to decay the recombination of e^−^/h^+^ pairs and improve the sonosensitization efficiency. Upon intravenous administration of CFAP in mouse models, tumor growth can be significantly inhibited without causing adverse side effects. Taken together, this study provides new insights on engineering bimetallic oxide Fenton agents for critical disease therapy, aiming at avoiding harsh reaction conditions and making the best use of TME.

## 4 Experimental Section

### Synthesis of CoFe_2_O_4_ Nanoflowers

Cobalt ferrite nanoflowers was synthesized through a facile hydrothermal process. Briefly, 360 mg FeCl_3_·6H_2_O, 190 mg CoCl_2_·6H_2_O, and 1.1 g polyvinylpyrrolidone (PVP) were added into 20 mL ethylene glycol solution. The mixture was under sonication for 30 min, followed by rigorously stirring till clear brown dispersion was obtained. Next, 850 mg CH_3_ONa was added into the above system, and the reaction was continued for 30 min under stirring to obtain clear black dispersion. Then, as‐prepared solution was placed in 50 mL Teflon liner, which was tightly sealed in a stainless‐steel embryo for reaction at 200 °C for 6 h. After cooling down to room temperature, the products were isolated through centrifugation at 10,000 rpm for 5 min and thoroughly washed with anhydrous ethanol for three times. At last, as‐synthesized CoFe_2_O_4_ nanoflowers were finally dispersed in deionized (DI) water and ready for use.

### Synthesis of CoFe_2_O_4_@Au

Au nanodots were growth onto the lattice structure of CoFe_2_O_4_ nanoflowers in situ. Briefly, 10 mL CoFe_2_O_4_ dispersion (4.7 mg mL^−1^) was agitated by sonication for 5 min. Then, 0.6 mL HAuCl_4_ (10 mm) was dropwise added into CoFe_2_O_4_ dispersion, and the mixture was under sonication for another 5 min. Afterward, 1.2 mL NaBH_4_ (10 mm) was dropwise added into the above system, and the mixture was under stirring in the dark for 4 h. Finally, CoFe_2_O_4_@Au was harvested through centrifugation at 10,000 rpm for 5 min and thoroughly washed with DI water for three times.

### Synthesis of CFAP

CFAP was prepared by one‐step surface PEGylation of CoFe_2_O_4_@Au. Briefly, 9 mL CoFe_2_O_4_@Au solution (1.7 mg mL^−1^) was agitated under sonication for 5 min. Subsequently, 1 mL PEG solution (1.53 mg mL^−1^) was added into CoFe_2_O_4_@Au dispersion, and the mixture was under mechanical stirring for 6 h. As‐developed CFAP was collected through centrifugation at 10,000 rpm for 5 min and thoroughly washed with DI water for three times. Lastly, the products were dried at in a vacuum drying oven at 60 °C for 12 h before further use. Loading content of Au in CFAP was determined from inductively coupled plasma mass spectrometry (ICP‐MS).

### Catalase (CAT)‐Mimic Activity of CFA

To investigate the CAT‐like activity, oxygen generation was measured at different concentrations of CFA (0, 100, 200, and 300 µg mL^−1^) in the presence of H_2_O_2_ (10 mm) at room temperature. The dissolved oxygen concentration was recorded from a portable oxygen sensing probe during the incubation for 7 min. Afterward, CAT‐like catalytic kinetics of CFA was characterized via classical Michaelis‐Mention equation. Briefly, the dissolved oxygen level in CFA dispersion (100 µg mL^−1^) was detected upon the addition of H_2_O_2_ at different concentrations (0, 10, 20, 40, and 80 mm) at room temperature for 5 min. The reaction rate (*v*) under different H_2_O_2_ concentrations was derived from the curve slope of H_2_O_2_ consumption over time. Michaelis‐Menten kinetic constant (K_M_) and maximum reaction velocity (V_max_) were quantified from the fitting data of Michaelis‐Menten saturation Equation (1).

(1) $$v=V_{\max}\times \left[S\right]/\left(K_{M}+\left[S\right]\right)$$

where [S] is the H_2_O_2_ concentration and *v* is the instantaneous reaction rate. The catalytic rate constant (K_cat_) could be calculated from Equation (2).

(2) $$K_{\mathrm{cat}}=\nu_{\max}/\left[E\right]$$

where K_cat_ is defined to be the rate constant of the maximum number of substrate molecules converted to products per unit time. [E] is the catalyst concentration. Catalytic efficiency was calculated in accordance with Equation (3).

(3) Catalyticefficiency=Kcat/KM

### Peroxidase (POD)‐Mimic Activity of CFA

POD‐mimic activity of CFA was evaluated by using different spectroscopic indicators, including o‐phenylenediamine (OPD), 3,3′,5,5′‐tetramethylbenzidine (TMB), 2,2′‐azino‐bis(3‐ethylbenzothiazoline‐6‐sulfonic acid) (ABTS), methylene blue (MB), and terephthalic acid (TA). Briefly, aqueous dispersion of CFA containing OPD (25 µg mL^−1^), TMB (125 µg mL^−1^), ABTS (25 µg mL^−1^), MB (10 µg mL^−1^), or TA (12.5 µg mL^−1^) was spectrophotometrically characterized after reaction for a specific period at pH 4 in the presence or absence of H_2_O_2_ (10 mm). The absorbance changes at 435 nm (OPD probe), 652 nm (TMB probe), 734 nm (ABTS probe), 665 nm (MB probe), and the fluorescence emission intensity at 426 nm (TA probe, λ_ex_: 315 nm) were measured to analyze the production of ·OH. Afterward, POD‐like catalytic kinetics of CFA was characterized via classical Michaelis‐Mention equation. Briefly, the absorbance change of TMB was measured during incubating CFA (200 µg mL^−1^) in different concentrations of H_2_O_2_ for 3 min. The reaction rates (*ν*) versus different concentration of H_2_O_2_ were calculated from the initial slope of OxTMB production. Michaelis‐Menten kinetic constant (K_M_) and maximum reaction velocity (V_max_) were quantified from the fitting data of Michaelis‐Menten saturation equation as previously described.

### Glucose Oxidase (GOD)‐Mimic Activity of CFA

GOD‐mimic activity of CFA was assessed through a dinitrosalicylic acid (DNS) method. Briefly, DNS stock solution was prepared by the addition of 1.26 g C_7_H_4_N_2_O_7_, 4.25 g NaOH, and 1 g phenol into 100 mL KNaC_4_O_6_ solution (0.364 g mL^−1^). To carry out the test, CFA (200 µg mL^−1^) was incubated in 0.5 mL glucose solution (1 mg mL^−1^) at 37 °C for 8 h. After the reaction was completed, 0.5 mL DNS solution was added into the above system, followed by reaction in a water bath at 100 °C for 8 min. After naturally cooling to room temperature, the supernatant was collected for spectrophotometric analysis.

In another aspect, ABTS^+·^ was used as the electron acceptor to detect the glucose oxidation. Briefly, ABTS^+·^ solution was prepared by mixing 1 mL ABTS (10 mm) with 1 mL K_2_S_2_O_8_ (3.5 mm), followed by storing in the dark for 12 h. In a typical test, the reaction system containing CFA (200 µg mL^−1^), ABTS^+·^ (0.1 mm), and glucose (50 mm) was incubated at 37 °C for 5 min. Finally, optical absorption spectra of supernatant were recorded through spectrophotometry.

### Electrochemical Test

The oxygen reductive reaction (ORR) performance of CFA was measured in 0.5 m Na_2_SO_4_ at room temperature. A rotating disk electrode (RRDE) was used to determine the number of reduced oxygen molecules on the electrode surface and further validate whether hydrogen peroxide was generated. Briefly, the working electrode was established by dropping 10 µL CFA (200 µg mL^−1^, in anhydrous ethanol) and 10 µL perfluorosulfonic acid polymer solution (Nafion solution) onto RRDE. Pt foil served as the counter electrode and Ag/AgCl electrode was utilized as the reference electrode. Before the electrochemical test, the electrolyte solution was purged with O_2_ or N_2_ for at least 30 min, aiming to create normoxia or hypoxia environment. The linear sweep voltammetry plots were recorded by applying the proper potential range (−1–0 V) at the scan rate of 10 mV s^−1^. The electron transfer number (n) and selectivity of CFA toward H_2_O_2_ production could be calculated according to Equations (4) and (5).

(4) $$n=4\;\frac{\left|I_{disk}\right|}{\left|I_{disk}\right|+I_{ring}/N}$$

(5) $$H_{2}O_{2}\;\%=200\;\frac{I_{ring}}{N\left|I_{disk}\right|+I_{ring}}$$

### US‐Triggered ^1^O_2_ Generation

Two commercial ^1^O_2_ probes, including 1, 3‐diphenyliso‐benzofuran (DPBF) and singlet oxygen sensor green (SOSG), were used for the detection of singlet oxygen. Briefly, 2 mL reaction system containing CFA (200 µg mL^−1^), SOSG (5 µm), and H_2_O_2_ (10 mm) was irradiated by a US source (1.0 MHz, 1.0 W cm^−2^, 50% duty cycle) for up to 5 min. Then, fluorescence spectra (λ_ex_/λ_em_ = 488/525 nm) were recorded from a fluorescence spectrometer. Alternatively, 2 mL reaction system containing CFA (50 µg mL^−1^), DPBF (10 µg mL^−1^), and H_2_O_2_ (10 mm) was irradiated by a US source (1.0 MHz, 1.0 W cm^−2^, 50% duty cycle) for up to 5 min. Lastly, the absorbance decay of supernatant at 417 nm was dynamically documented through spectrophotometry.

### Electron Spin Resonance (ESR) Spectroscopy

To category ROS, ESR spectroscopy was carried out by using the spin traps of 5,5‐dimethylpyrroline N‐oxide (DMPO) and 2,2,6,6‐tetramethylpiperidine (TEMP) to detect ·OH and ^1^O_2_ radicals, respectively. To probe ·OH radicals, ESR spectrum of CFA (400 µg mL^−1^) containing H_2_O_2_ (10 mm) was measured from a ESR spectrometer after reaction in the presence of DMPO (25 mg mL^−1^) at pH 4 for 30 min. As for ^1^O_2_ sensing, ESR spectrum of CFA (400 µg mL^−1^) was recorded in the presence of TEMP (400 mm) after US irradiation source (1.0 MHz, 1.0 W cm^−2^, 50% duty cycle) for 5 min. The waveforms were analyzed to convey the ROS information.

### Intracellular ROS Detection

Fluorescent probe of 2′7’‐dichlorofluorescin diacetate (DCFH‐DA, λ_ex_/λ_em_ = 488/525 nm) was experimentally used for detection the intracellular ROS level. Briefly, 4T1 cells were seeded in a 12‐well plate (5 × 10^4^ cells per well) and cultured at 37 °C for 12 h. Afterward, these cells were administered with CFAP (75 µg mL^−1^) for 6 h, followed by US irradiation (1.0 MHz, 0.5 W cm^−2^, 20% duty cycle) for 5 min. After incubation for another 2 h, the cells were stained with DAPI (0.4 µg mL^−1^) for 25 min and DCFH‐DA (10 µm) for 20 min. At last, fluorescence emission from cytosolic region was visualized and analyzed through confocal microscopy.

### Cytotoxicity In Vitro

Cytotoxic effect of CFAP on 4T1 tumor cells in vitro were studied through standard MTT assay. Briefly, 4T1 cells were seeded in a 96‐well plate (1 × 10^4^ cells per well) and cultured at 37 °C for 12 h, followed by incubation with CFAP at different concentrations (0, 18, 37, 75, 150, 300 µg mL^−1^) for 6 h. Thereafter, the cells were irradiated by a US source (1.0 MHz, 0.5 W cm^−2^, 20% duty cycle) for 5 min. At last, cell viability in each well was quantified by MTT assay as previously described. To explore the ferroptosis level, ferrostatin‐1 (Fer‐1, 70 µg mL^−1^) was introduced in all the previous administration groups, followed by the quantification of cell viability.

### Live/Dead Cell Differential Staining

Inhibition of tumor cell growth by CFAP was further assessed by live/dead cell differential staining assay. Briefly, 4T1 cells were seeded in a 12‐well plate (2 × 10^5^ cells per well) and cultured at 37 °C for 12 h, followed by treatment with CFAP (150 µg mL^−1^) for 6 h. Then, the cells received US irradiation (1.0 MHz, 0.5 W cm^−2^, 20% duty cycle) for 5 min. After culture for another 18 h, the treated cells were co‐stained with fluorescein diacetate (FDA) and propidium iodide (PI) for 15 min, followed by examination through fluorescence microscopy.

### Intracellular Oxygen Level Detection

To simulate hypoxic TME, 4T1 cells in a 12‐well plate (5 × 10^4^ cells per well) were cultured in a hypoxic atmosphere (1% O_2_ and 5% CO_2_) at 37 °C for 12 h. Then, the cells were treated with CFAP (75 µg mL^−1^) in the presence of [Ru(dpp)_3_]Cl_2_ (3.75 µm, λ_ex_/λ_em_ = 455/613 nm) for 10 h, followed by US irradiation (1.0 MHz, 0.5 W cm^−2^, 20% duty cycle) for 5 min. After incubation for another 2 h, the cells were stained with DAPI (0.4 µg mL^−1^) for 25 min, followed by examination through confocal microscopy.

### Mitochondrial Damage Evaluation

Mitochondrial health status after CFAP treatment was assessed by monitoring mitochondrial membrane potential (MMP). Briefly, 4T1 cells in a 12‐well plate (5 × 10^4^ cells per well) were cultured at 37 °C for 12 h. Next, the cells were exposed to CFAP (75 µg mL^−1^) for 6 h, followed by US irradiation (1.0 MHz, 0.5 W cm^−2^, 20% duty cycle) for 5 min. After incubation for another 2 h, the treated cells were sequentially stained with JC‐1 dye (20 µg mL^−1^) for 15 min and DAPI (0.4 µg mL^−1^) for 25 min. Finally, fluorescence emission from cytosolic region was visualized through confocal microscopy.

### Lipid Peroxidation Assay

Lipid peroxidation caused by CFAP was quantified by malondialdehyde (MDA) assay kit. 4T1 cells were seeded in a 6‐well plate (2 × 10^5^ cells per well) and cultured at 37 °C for 12 h. Subsequently, the cells were exposed to CFAP (75 µg mL^−1^) for 6 h, followed by US irradiation (1.0 MHz, 0.5 W cm^−2^, 20% duty cycle) for 5 min. After incubation for another 2 h, all the treated cells were resuspended in thiobarbituric acid (TBA) solution, which were further placed in the atmosphere at 100 °C for 15 min and immediately cooled in an ice bath for 10 min. Subsequent to the centrifugation at 1,000 g for 10 min, the absorbance of supernatant was measured at the wavelength of 532 nm through spectrophotometry. Finally, intracellular MDA level was quantified from the corresponding standard curve.

### Apoptosis Assessment

Specifically, 4T1 cells were seeded in a 12‐well plate (1 × 10^5^ cells per well) and cultured at 37 °C for 12 h. Subsequently, the cells were exposed to CFAP (200 µg mL^−1^) for 8 h, followed by US irradiation (1.0 MHz, 0.5 W cm^−2^, 20% duty cycle) for 5 min. After incubation for another 4 h, the cells were trypsinized (without EDTA chelation) and resuspended in PBS containing Annexin V‐FITC and PI. After staining for 20 min in the dark, cytosolic fluorescence was detected and analyzed through flow cytometry.

### Scratch Assay

Anti‐migration capacity of CFAP toward 4T1 cells was investigated through scratch assay. Briefly, 4T1 cells were seeded in a 24‐well plate (1 × 10^5^ cells per well) and cultured at 37 °C for 12 h to reach confluence. Next, a straight scratch (≈0.7 mm in width) was created by using a 200 µL pipette tip. Then, the cells received the treatments similar with those in cytotoxicity assay. After incubation for 6, 12, 24, and 48 h, scratch healing was inspected under an inverted microscope.

### Expression Levels of Metastasis‐Associated Proteins

The expressive levels of metastasis‐associated proteins, including matrix metalloproteinase‐9 (MMP‐9), vascular endothelial growth factor (VEGF) and E‐cadherin, were measured in 4T1 cells after treatment with CFAP. Briefly, after receiving the treatments similar with those in analyzing DAMPs release, the treated cells were washed with PBS and fixed with paraformaldehyde (4%) for 20 min. Then, the cells were permeated with Triton X‐100 (0.1%) for 5 min and blocked with bovine serum albumin (BSA, 1%) at room temperature for 1 h. Subsequently, the cells were exposed to the primary antibodies of anti‐MMP‐9, anti‐VEGF and anti‐E‐cadherin (2 µg mL^−1^) at 4 °C for 12 h, followed by labeling with FITC‐labeled goat anti‐rabbit lgG secondary antibodies (4 µg mL^−1^) at 4 °C for 4 h. Lastly, the cells were stained with DAPI (in anti‐quenching sealing agent) for 25 min and examined through confocal microscopy.

### Tumor Model Establishment

Animal experiments were authorized by the Institutional Animal Care and Use Committee (IACUC) of Southwest University, China (Approval number: IACUC‐20231121‐01), and conducted strictly complying with the National Guidelines for the Care and Use of Experimental Animals (China). To establish mouse tumor model, 100 µL 4T1 cells (5 × 10^5^ cell per mL, in saline) were subcutaneously inoculated into the right flank of female BALB/c mice (6 weeks, ≈18 g), followed by housing for about one week. As the tumor size reached ≈200 mm^3^, the mice were qualified for following antitumor evaluation in vivo.

### Tumor Eradication In Vivo

All the tumor‐bearing mice were allocated into the following six groups (*n* = 5 in each group), including 1) Saline, 2) US, 3) CFP, 4) CFAP, 5) CFP + US, and 6) CFAP + US. On days 0 and 3, all the agents (25 mg kg^−1^, in saline) were intravenously injected into the tail vein of mice. On days 1 and 4, US irradiation (1.0 MHz, 1.0 W cm^−2^, 50% duty cycle) was implemented against the tumor region for 5 min where applicable. Tumor volume and mouse body weight were recorded daily during the treatment period of two weeks. As the treatment ended, all the mice were sacrificed to harvest tumor and major organs (heart, liver, spleen, lung, and kidney). Tumor growth inhibition (TGI) index was determined from the following Equation (6).

(6) TGI=(VC−VT)/VC×100%

where V_C_ and V_T_ signify the tumor size of saline and other experimental groups, respectively. Pathological status of tumor tissue was analyzed through versatile staining of histological sections with hematoxylin & eosin (H&E), terminal deoxynucleotidyl transferase dUTP nick end labeling (TUNEL), Ki67, CRT, and HMGB1.

### Metastasis Suppression In Vivo

Liver metastasis suppression by CFAP was studied by using BALB/c mice‐bearing 4T1 tumors. Specifically, all the mice were randomly allocated into the following three groups (*n* = 5 in each group), including 1) Saline, 2) CFAP, and 3) CFAP + US. On days 0 and 3, 100 µL CFAP dispersion (25 mg kg^−1^, in saline) was intravenously injected into the mouse caudal vein. US irradiation (1.0 MHz, 1.0 W cm^−2^, 50% duty cycle) was carried out against the tumor region for 5 min at 24 h after each intravenous administration. On day 8, 100 µL 4T1 cells dispersion (1 × 10^7^ cells per mL, in saline) was intravenously injected into the tumor‐bearing mice via the tail vein. On day 21, liver tissue was dissected for photographic documentation. Histopathological characteristics were analyzed by staining with H&E and Oct4.

### Statistical Analysis

Data were displayed as mean ± standard deviation (SD, *n* = 4 unless otherwise declared). The error bars signify the SD from independent assayed samples. Statistical analysis was implemented with GraphPad Prism 8.0 (GraphPad Software, USA). Unpaired two‐tailed Student's t‐test was performed to determine the significant difference between two groups. ^*^ *p* < 0.05, ^**^ *p* < 0.01, ^***^ *p* < 0.001.

## Conflict of Interest

The authors declare no conflict of interest.

## Supporting information

Supporting Information

## Data Availability

The data that support the findings of this study are available from the corresponding author upon reasonable request.
