# Supplementary material for: Gold Nanodots‐Anchored Cobalt Ferrite Nanoflowers as Versatile Tumor Microenvironment Modulators for Reinforced Redox Dyshomeostasis
Source: Adv Sci (Weinh). 2024 Jul 10;11(34):2406683. doi: 10.1002/advs.202406683 (PMC11529044; doi:10.1002/advs.202406683)
Supplement: Supplementary file 1 — Supporting Information [file ADVS-11-2406683-s001.docx]

Supporting Information

**Gold nanodots-anchored cobalt ferrite nanoflowers as versatile tumor microenvironment modulators for reinforced redox dyshomeostasis**

*Guicheng Zeng, Jinning Mao, Haiyan Xing, Zhigang Xu, Zhong Cao, Yuejun Kang, Guodong Liu^*^, Peng Xue^*^*

G. Zeng, H. Xing, Z. Xu, Y. Kang, P. Xue

School of Materials and Energy

Southwest University

Chongqing 400715, China

E-mail: [xuepeng@swu.edu.cn](mailto:xuepeng@swu.edu.cn) (P. Xue)

J. Mao

Health Management Center

The Second Affiliated Hospital of Chongqing Medical University

Chongqing, 400016, China

Z. Cao

School of Biomedical Engineering

Shenzhen Campus of Sun Yat-sen University

Shenzhen, Guangdong 518107, China

G. Liu

Department of Neurosurgery

The Second Affiliated Hospital of Chongqing Medical University

Chongqing, 400016, China.

Email: [304678@hospital.cqmu.edu.cn](mailto:304678@hospital.cqmu.edu.cn) (G. Liu)

G. Zeng and J. Mao contributed equally to this work.

Keywords: cobalt ferrite, nanocatalysts, reactive oxygen species, sonodynamic therapy, tumor microenvironment

**Supplementary methods**

***Materials and reagents:*** Porphyrin monomethyl ether (HMME, 99%), manganese chloride tetrahydrate (MnCl_2_·4H_2_O, >99.9%), nitric acid (HNO_3_, 42.24%), NaOH (> 98%), ethyl alcohol, o-phenylenediamine (OPD, >99.9%), hyaluronan (HA, MW: 10 kDa), L-α-phosphatidylglycerol (egg, chicken, >99%), agar (ash, <1.5%) and chloral hydrate (99%) were obtained from Sigma-Aldrich (USA). Fluorescein diacetate (FDA, >95%), propidium iodide (PI, >94%), N-hydroxysuccinimide (NHS, >98%), 1-ethyl-3-(3-dimethylaminopropyl) carbodiimide (EDC, >98%), 1,3-diphenylisobenzofuran (DPBF), methylene blue (MB, 0.05 wt% in H_2_O), hydrogen peroxide (H_2_O_2_, 30 wt% in H_2_O), crystal violet (0.5 wt% in H_2_O) and hyaluronidase (HAase, ≥300 IU mg^-1^) were purchase from Shanghai Aladdin Bio-Chem Technology Co., Ltd. (China). MTT cell viability assay kit, fetal bovine serum (FBS), phosphate-buffered saline (PBS, pH 7.4), 5,5’-dithio-bis-(2-nitrobenzoic acid) (DTNB, 98%) were supplied by Shanghai Macklin Biochemical Co., Ltd. (China). 5,5-dimethyl-1-pyrroline N-oxide (DMPO, >97%), 2,2,6,6-tetramethyl-4-piperidone (TEMP, >98%) and Singlet Oxygen Sensor Green (SOSG) sensor were ordered from Dalian Meilunbio Co., Ltd (China). Dulbecco’s modified eagle’s medium (DMEM), 4',6-diamidino-2-phenylindole (DAPI, 98%) and Hoechst 33342 were acquired from Thermo Fisher Scientific (USA). 2’,7’-dichlorofluorescin diacetate (DCFH-DA, >97%), dimethyl sulfoxide (DMSO, >99.9%), BCA protein assay kit, luminescent ATP enhanced detection assay kit and radio immunoprecipitation assay (RIPA) lysis buffer were provided by Beyotime Biotechnology (China). BALB/c mice were supplied by Chongqing Tengxin Bill Experimental Animal Sales Co., Ltd. (China). L929 mouse fibroblasts (L929s), human umbilical vein endothelial cells (HUVEC) and RAW264.7 cells were provided by the Cell Bank of Type Culture Collection of Chinese Academy of Sciences (Shanghai, China). Deionized (DI) water (18.2 MΩ cm) was collected from a Milli-Q Synthesis A10 purification system (Molsheim, France).

***Characterizations:*** Morphological features of nanoparticles were examined through transmission electron microscopy (TEM; LIBRA®120 PLUS, Carl Zeiss, Germany) and field emission scanning electron microscopy (FESEM; JSM-7800F, JEOL, Japan). Atomic force microscopic (AFM) images were taken from Bruker Dimension Icon microscope (Bruker, Germany). Hydrodynamic size and zeta potential were measured by a Zetasizer Nano ZS90 analyzer (Malvern Panalytical, UK). Crystallographic form was analyzed by X-ray powder diffraction (XRD) through an XRD-7000 X-ray diffractometer (Shimadzu, Japan) with CuKα radiation (λ = 1.5406 Å). Ultraviolet-visible (UV-Vis) absorption spectra were acquired from Shimadzu UV-1800 UV/Visible Scanning Spectrophotometer (Shimadzu, Japan). Fluorescence spectra were measured from a RF-5301 spectrofluorometer (Shimadzu, Japan). 3D fluorescence spectra were recorded from Duetta fluorescence and absorbance spectrometer (HORIBA Scientific, Japan). Elemental composition of products was determined by X-ray photoelectron spectroscopy (XPS) from an ESCALAB 250Xi XPS spectrometer (Thermo Fisher Scientific, USA). Electron spin resonance (ESR) spectra were measured by using an EMX Nano spectrometer (Bruker, USA). Raman spectra were recorded from a LabRam HR800 Raman spectrometer (Horiba, Japan). Fluorescence images of bacterial and biofilm were taken from a confocal microscope (LSM800, Zeiss, Germany).

***Biocompatibility in vitro:*** To investigate the biocompatibility of CFAP on normal somatic cells, L929 murine fibroblasts (L929s) and human umbilical vein endothelial cells (HUVECs) were selected for evaluation based on 3-(4,5-dimethylthiazol-2-yl)-2,5-diphenyltetrazolium bromide (MTT) assay. Briefly, cells were seeded in a 96-well plate (1 × 10^4^ cells per well) and cultured at 37 ℃ for 12 h. Then, the cells were treated with different concentrations of CFAP (0, 18, 37, 75, 150, 300 μg mL^-1^) for 24 h. After rinsing with PBS, these cells were exposed to 3-(4, 5-dimethylthiazol-2-yl)-2, 5-diphenyltetrazolium bromide (MTT) for another 4 h. Then, the purple crystals of methylzan were dissolved by dimethyl sulfoxide (DMSO) after oscillation for 15 min. At last, optical absorbance in each well was measured at the wavelength of 490 nm through a microplate reader, and the cell viability was determined from the manufacturer’s protocol.

***Hemocompatibility in vitro:*** Blood compatibility of CFAP *in vitro* was evaluated by detecting the hemolysis rate of red blood cells (RBCs) after contact. Briefly, whole blood was withdrawn from the venous sinus of mouse orbit and RBCs were harvested through centrifugation at 3,000 rpm for 5 min. Afterwards, RBCs (4% v/v, in PBS) were treated with different concentrations of CFAP (0, 31.3, 62.5, 125, 250, 500 μg mL^-1^) at 37 ℃ for 6 h. RBCs in PBS and DI water served as the negative and positive references, respectively. After centrifugation at 10,000 rpm for 5 min, supernatant was collected for spectrophotometric analysis. As the absorbance at 570 nm was acquired, the hemolysis rate was calculated according to the following equation (1).

Hemolysis rate (%) = (A-A_0_) / (A_∞_-A_0_) × 100% (1)

Where A, A_0_ and A_∞_ are the supernatant absorbance for the groups of CFAP, PBS and DI water, respectively.

***Cellular uptake in vitro:*** To investigate the endocytosis of CFAP by tumor cells, both confocal microscopy and flow cytometry were used for characterizations. First, CFAP was labeled with fluorescein isothiocyanate (FITC) through covalent conjugation. Briefly, 1 mL CFAP (0.5 mg mL^-1^) was reacted with FITC (0.5 mg mL^-1^) upon EDC/NHS activation in the dark for 12 h. Then, FITC-labeled CFAP was magnetically isolated and rinse with DI water to eliminate free FITC molecules. To study the cellular uptake, 4T1 cells were seeded in a 6-well plate (2 × 10^5^ cells per well) and cultured at 37 ℃ for 12 h. Subsequently, the adherent cells were exposed to CFAP (150 μg mL^-1^) for 0.5, 1, 2, 4 and 6 h. Afterwards, cells nuclei were stained with DAPI (1 μg mL^-1^) for 25 min, followed by examination through confocal microscopy. Regarding to flow cytometry, these treated cells were trypsinized and resuspended in PBS containing Ca^2+^/Mg^2+^ prior to cytometric analysis.

***Western blot.*** To extract cell protein contents, 4T1 cells were lysed in RIPA lysis buffer containing protease inhibitor PMSF (1%) on ice. After complete cell lysis, supernatant was harvested through centrifugation at 12,000 rpm at 4 ℃ for 10 min. Subsequently, the protein contents were determined through BCA protein assay kit. The lysates were thereafter loaded in sodium dodecyl sulfate polyacrylamide (SDS-PAGE) gel (12%), which was then transferred to a poly (vinylidene difluoride) (PVDF) membrane. The membrane was blocked with 5% bovine serum albumin at 25 ℃ for 2 h and incubated with primary antibodies at 4 ℃ for 12 h. After thoroughly washing with TBST solution, the membrane was incubated with enzyme-labeled secondary antibodies at room temperature for 2 h. At last, immunoblotting was examined *via* Western-Ready enhanced chemiluminescence (ECL) substrate detection kit.

***DAMPs release in vitro:*** Therapy-induced release of representative DAMPs from tumor cells, including calreticulin (CRT), high-mobility group box 1 protein (HMGB1) and adenosine-triphosphate (ATP), were investigated *in vitro*. Briefly, 4T1 cells were seeded in a 12-well plate (5 × 10^4^ cells per well) and cultured at 37 ℃ for 12 h. Subsequently, the cells were exposed to CFAP (75 μg mL^-1^) for 6 h, followed by US irradiation (1.0 MHz, 0.5 W cm^-2^, 20% duty cycle) for 5 min. After incubation for another 2 h, the treated cells were washed with PBS and fixed with paraformaldehyde (4 %) for 20 min. Then, the cells were permeated with Triton X-100 (0.1%) for 5 min and blocked with bovine serum albumin (BSA, 1%) at room temperature for 1 h. Subsequently, the cells were exposed to the primary antibodies of anti-CRT (2 μg mL^-1^) and anti-HMGB1 (2 μg mL^-1^) at 4 ℃ for 12 h, followed by labeling with FITC-labeled goat anti-rabbit lgG secondary antibodies (4 μg mL^-1^) at 4 ℃ for 4 h. Next, the cells were stained with DAPI (in anti-quenching sealing agent) for 25 min and examined through confocal microscopy. In another aspect, ATP contents in cytosolic region before and after treatment was monitored by ATP assay kit. Briefly, after various treatments, the treated cells were lysed in ice-cold ATP detection buffer for 30 min, followed by collecting supernatant through centrifugation at 13,000 rpm at 4 °C for 10 min. The chemiluminescence of supernatant was measured through spectrophotometry. Finally, intracellular or extracellular ATP level was determined from the corresponding chemiluminescence standard curve.

***Complete blood count:*** BALB/c mice were intravenously injected with 100 μL CFAP (25 mg kg^-1^, in saline) *via* the tail vein. On day 0, 1, 3, 5, 7 and 14, whole blood was sampled from the retrobulbar venous plexus of mice. Thereafter, key blood indicators, including white blood cells (WBC), lymphocytes (LY), monocytes (MNC), red blood cells (RBC), hemoglobin (HGB), granulocytes (GR), platelets (PLT), mean corpuscular volume (MCV), hematocrit (HCT), mean corpuscular hemoglobin (MCH), mean platelet volume (MPV) and mean corpuscular hemoglobin concentration (MCHC), were analyzed from an automatic hematology analyzer.

**Supplementary data**


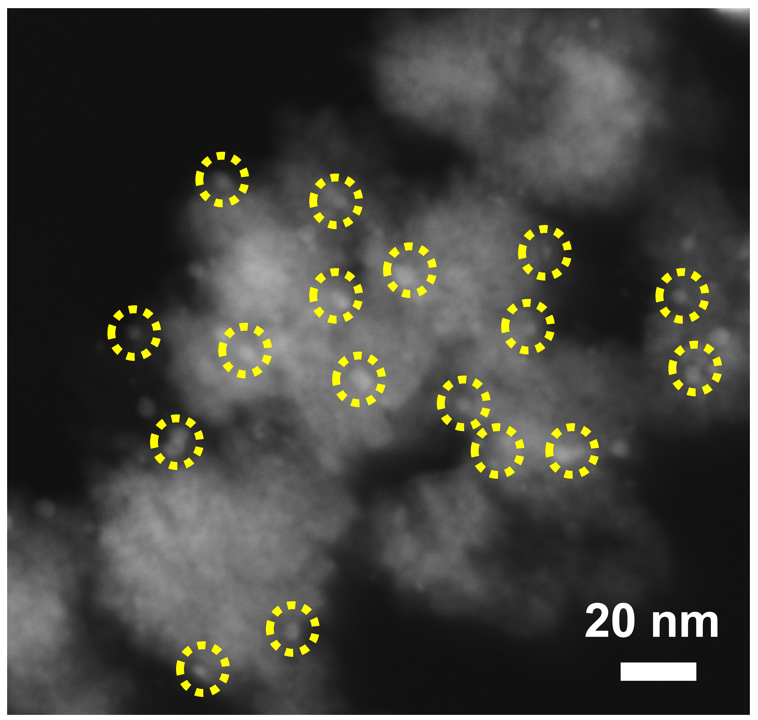


**Figure S1.** HAADF-STEM image of CFAP. Dashed yellow cycles donate tiny Au nanodots.


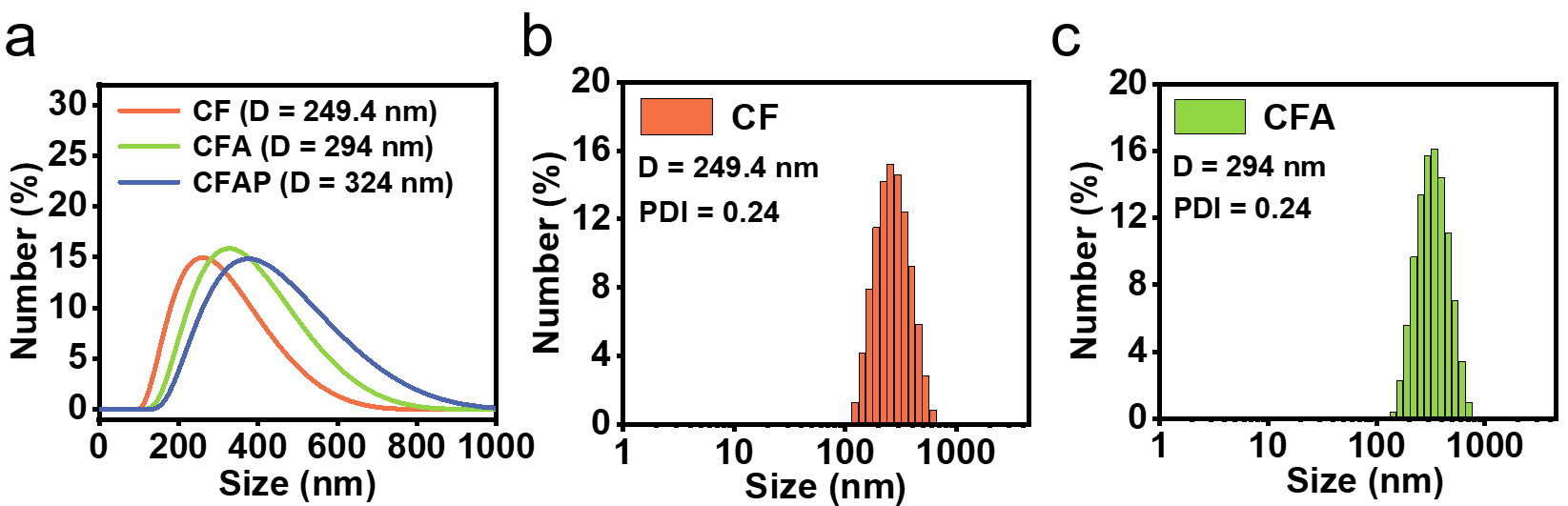


**Figure S2.** Hydrodynamic sizes of CF, CFA and CFAP measured by DLS. (a) Curve chart depiction. Histograms of (b) CF and (c) CFA.


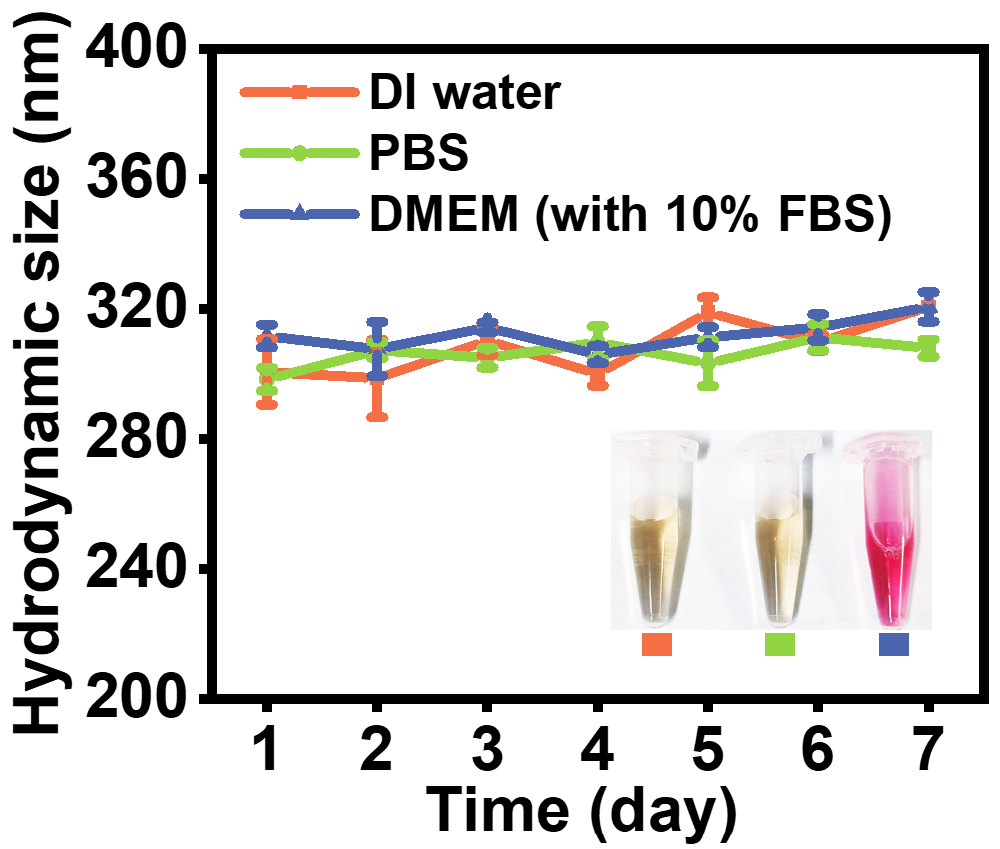


**Figure S3.** Hydrodynamic size of BAC during the incubation in DI water, PBS or DMEM (with 10% FBS) for seven days. Data are displayed as mean ± SD (n = 4).


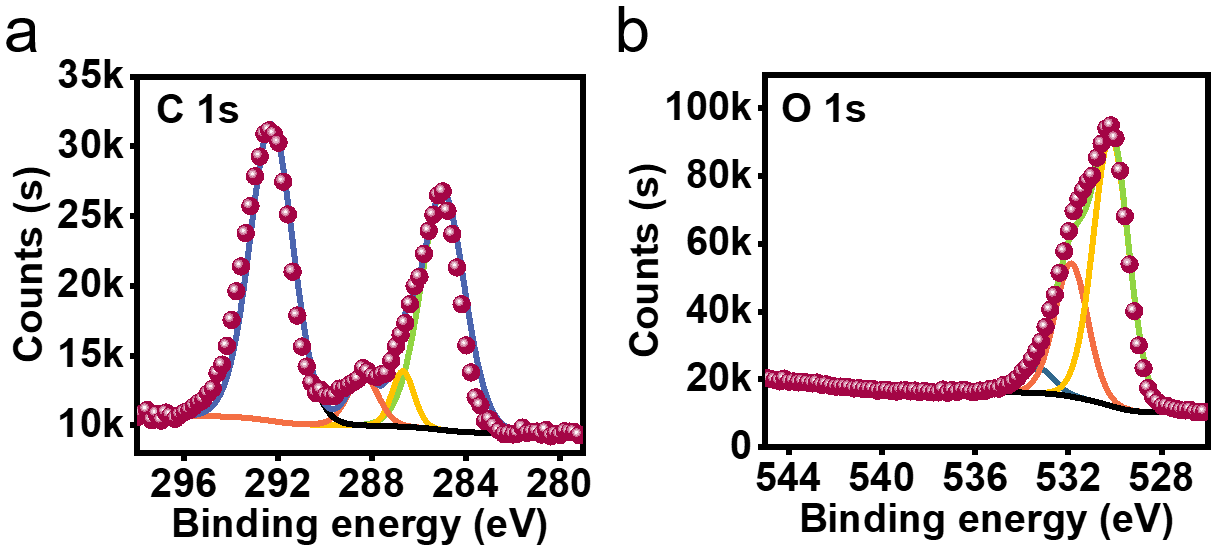


**Figure S4.** Core-level XPS spectra of (a) C 1s and (b) O 1s in CFAP.


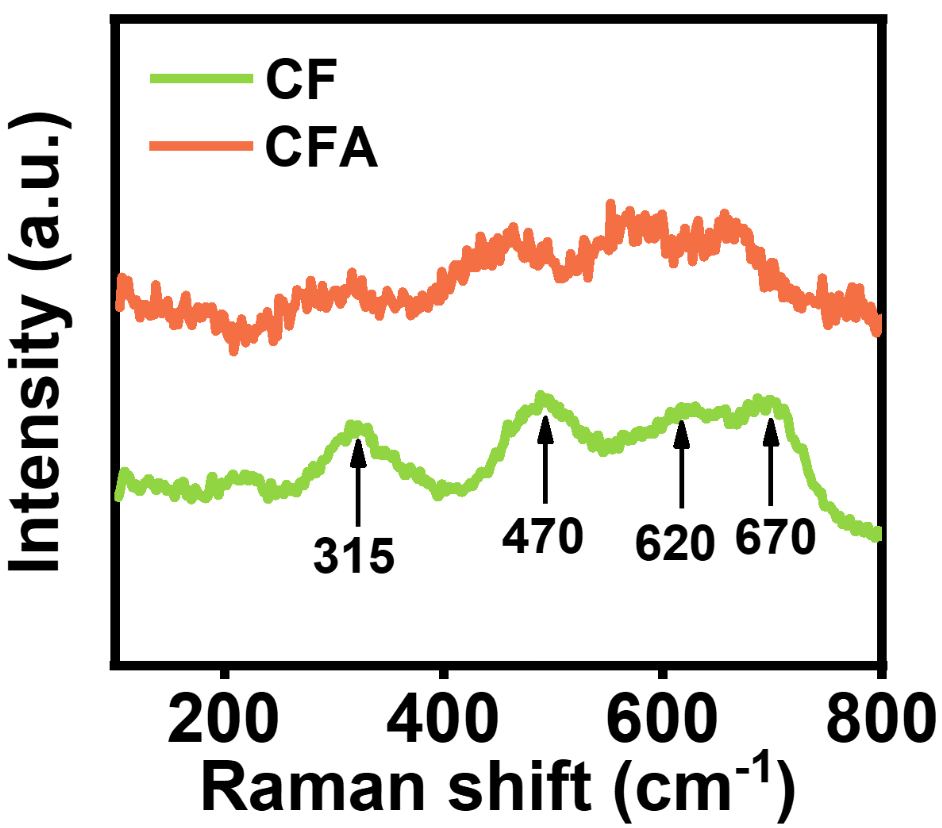


**Figure S5.** Raman spectra of CF and CFA.


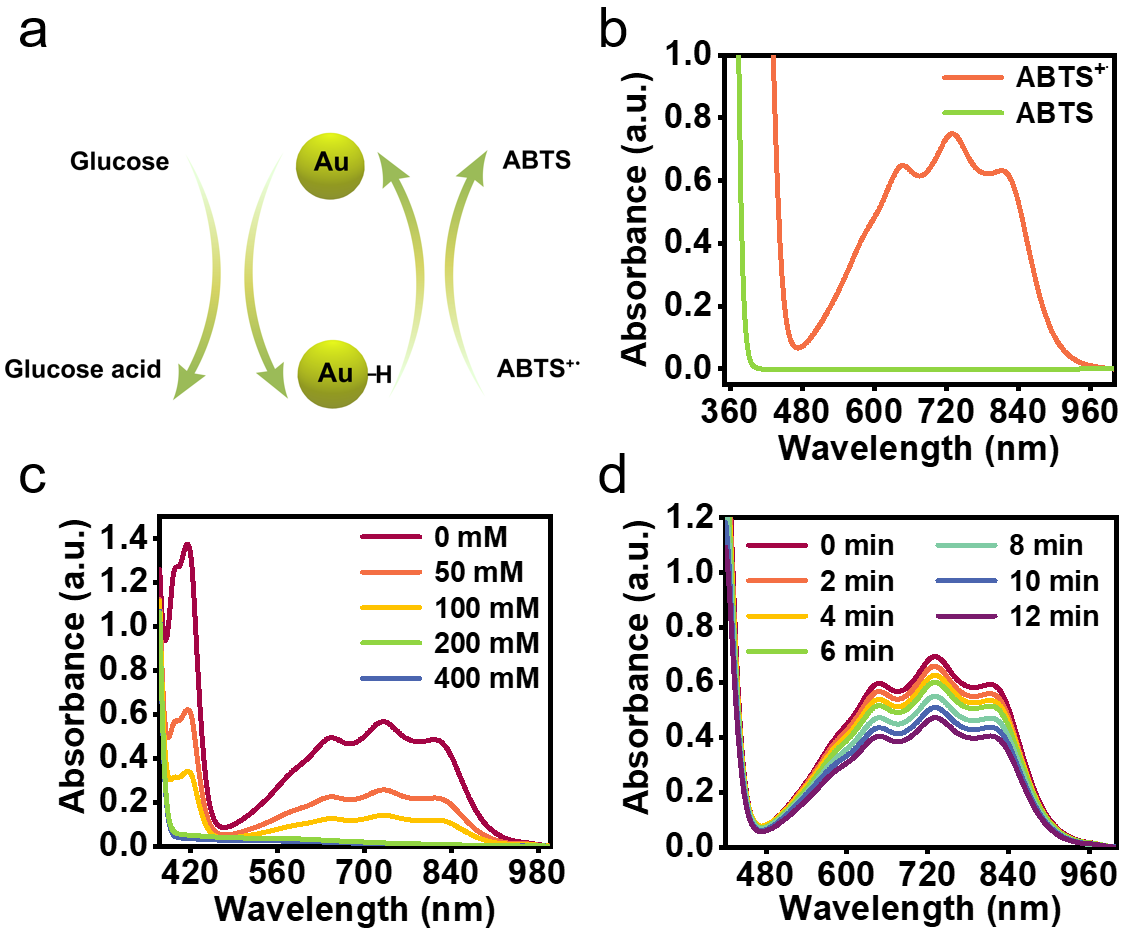


**Figure S6.** Glucose consumption by CFA. (a) Working mechanism of ABTS^+∙^ for the detection of glucose consumption. (b) Optical absorption spectra of ABTS and ABTS^+∙^. (c) Optical absorption spectra of ABTS^+∙^ containing CFA (200 μg mL^-1^) in the presence of glucose at different concentrations (0-400 mM) after reaction for 20 min. (d) Optical absorption spectra of ABTS^+∙^ containing CFA (200 μg mL^-1^) in the presence of glucose (50 mM) after reaction for various periods (0-12 min).


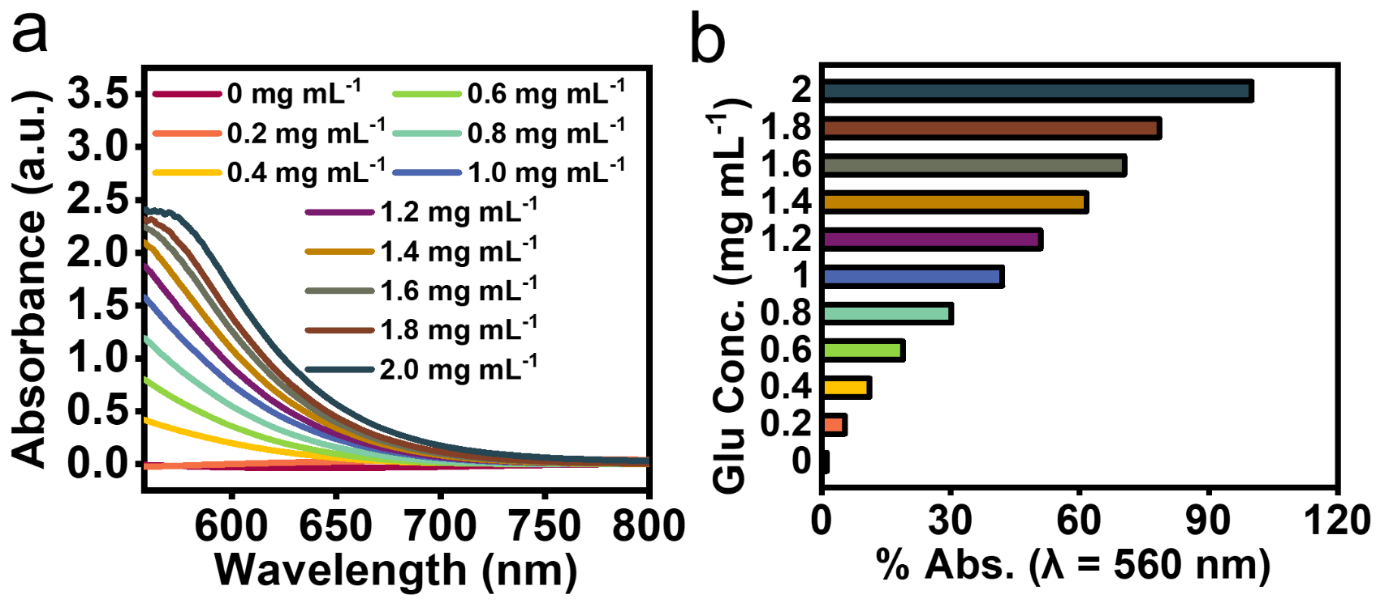


**Figure S7.** (a) Optical absorption spectrum of DNS containing CFA (50 μg mL^-1^) in the presence of glucose at different concentrations (0-2 mg mL^-1^) after reaction for 8 h. (b) Quantification of relative absorbance at 560 nm corresponding to panel (a).


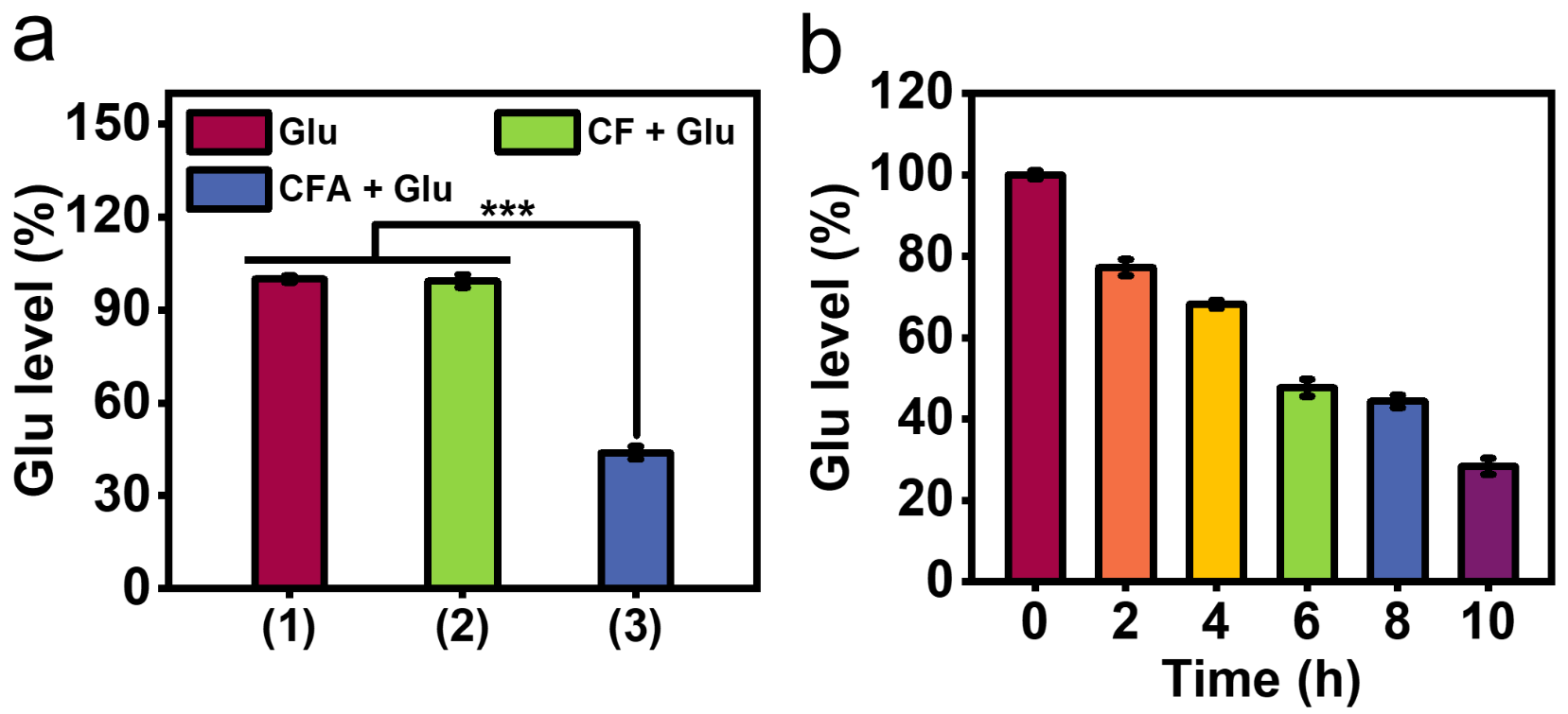


**Figure S8.** (a) Relative glucose level after treatment with CF or CFA for 8 h measured by DNS. (b) Relative glucose level after treatment with CFA (200 μg mL^-1^) after incubation for various periods (0-10 h). Data are displayed as mean ± SD (n = 4). ^***^*p* < 0.001.


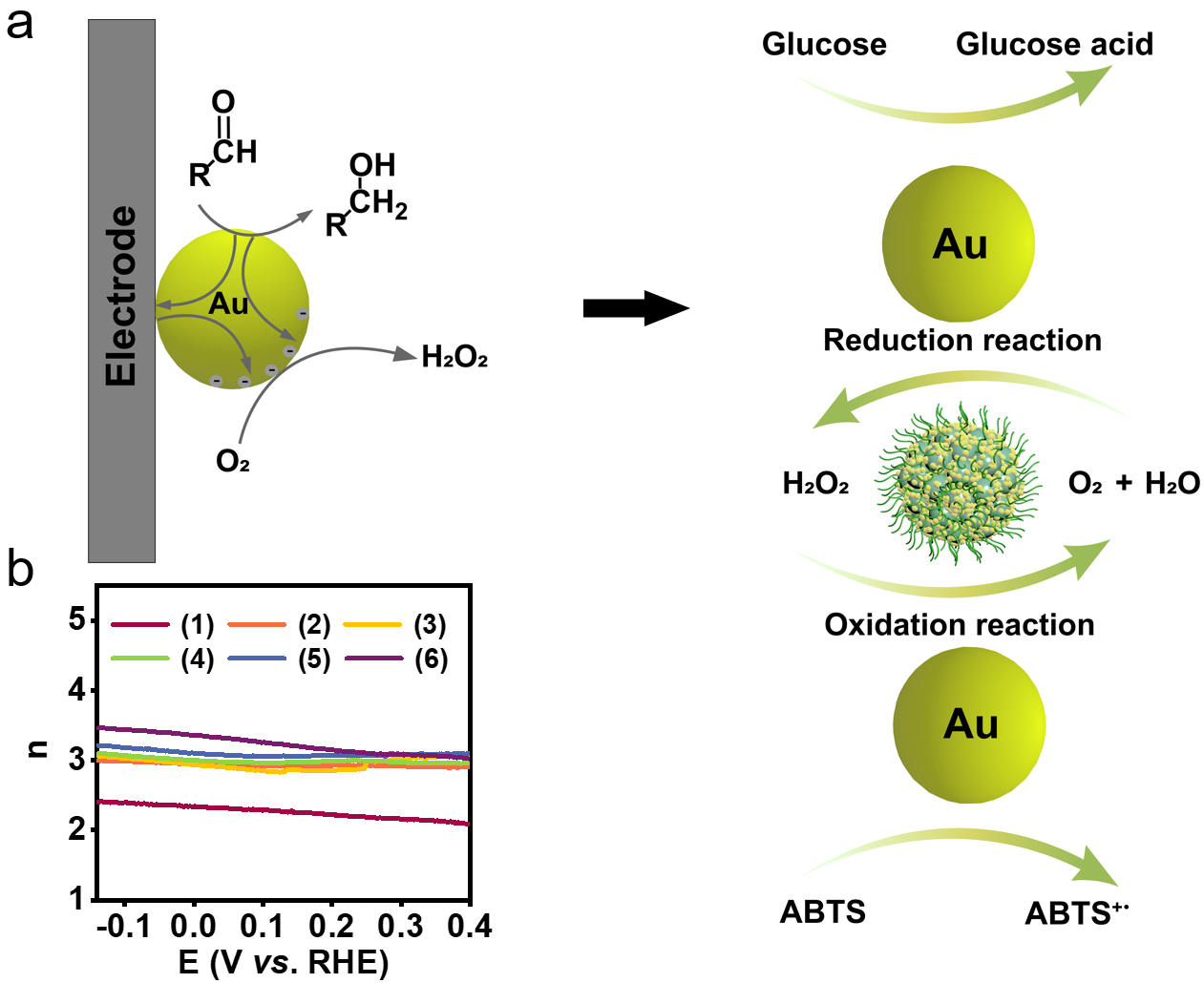


**Figure S9.** RRDE measurement for glucose consumption. (a) Schematic diagram to illustrate the electron transfer during oxygen reduction on RRDE in the presence of CFA. (b) Relative number of transfer electrons in terms of different treatment groups. Groups are allocated to be (1) CFA + Glu+ O_2_, (2) CFA + O_2_, (3) O_2_, (4) CFA + Glu+ N_2_, (5) CFA + N_2_, (6) N_2_.


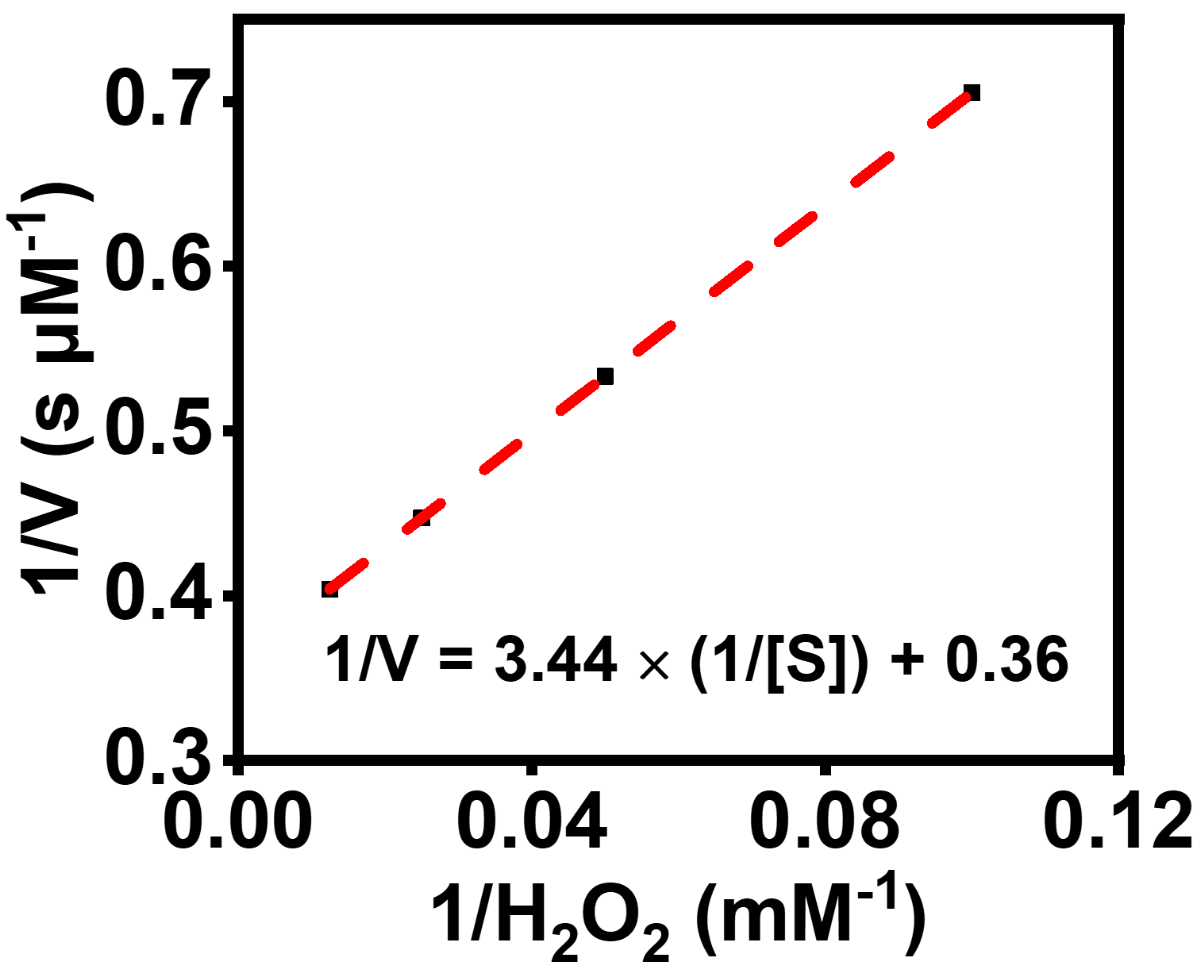


**Figure S10.** Double-reciprocal plot generated from Figure 3d.


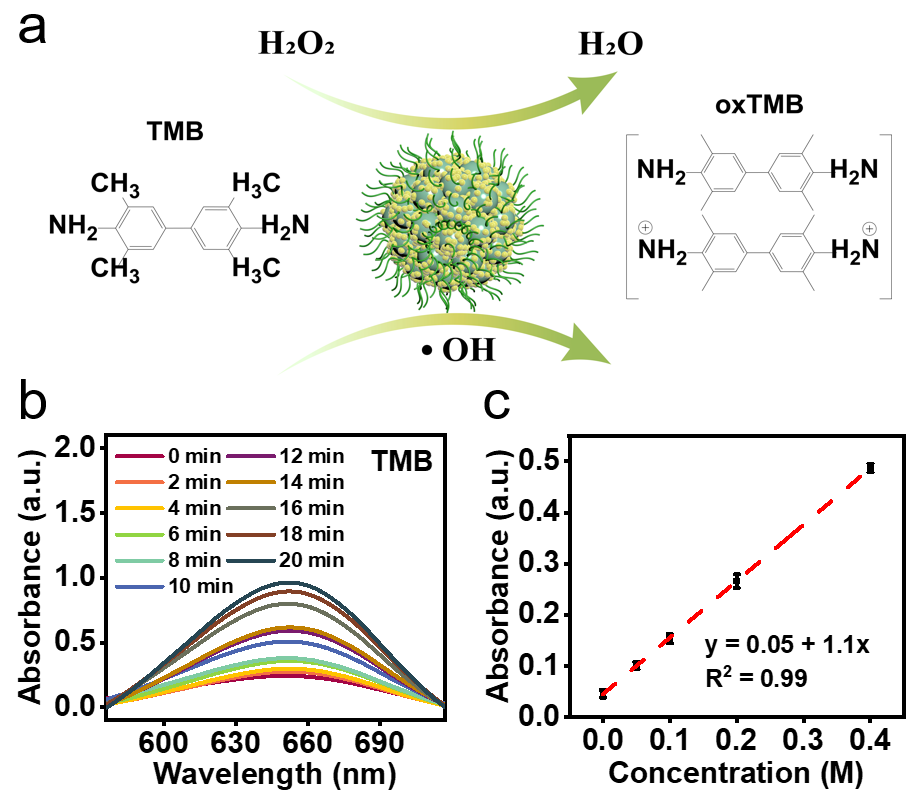


**Figure S11.** (a) Schematic diagram to illustrate the detection mechanism of ·OH by using TMB. (b) Optical absorption spectra of TMB (125 μg mL^-1^) containing CFA (60 μg mL^-1^) in the presence of H_2_O_2_ at different doses (10 mM) after reaction for different periods (0-20 min). (c) Calibration curve of Abs. (λ = 652 nm) *vs.* HRP concentration.


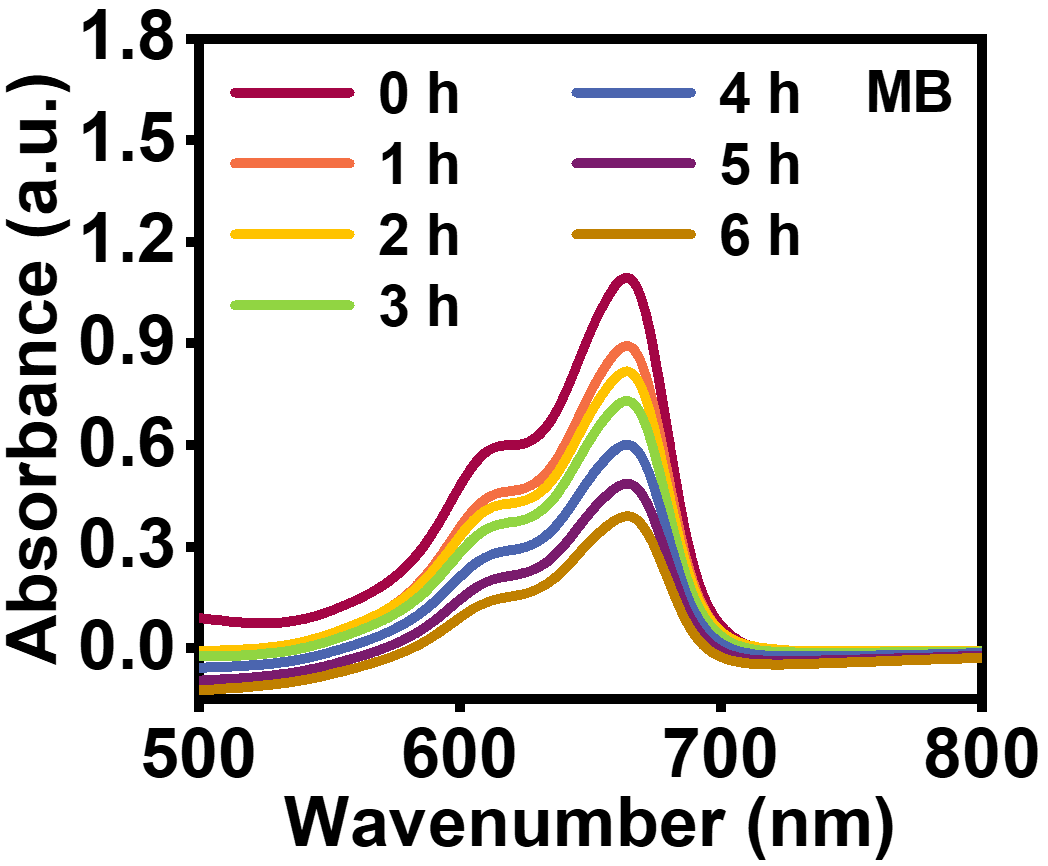


**Figure S12.**Optical absorption spectra of MB (10 μg mL^-1^) containing CFA (50 μg mL^-1^) and H_2_O_2_ (10 mM) after reaction for various periods (0-6 h).


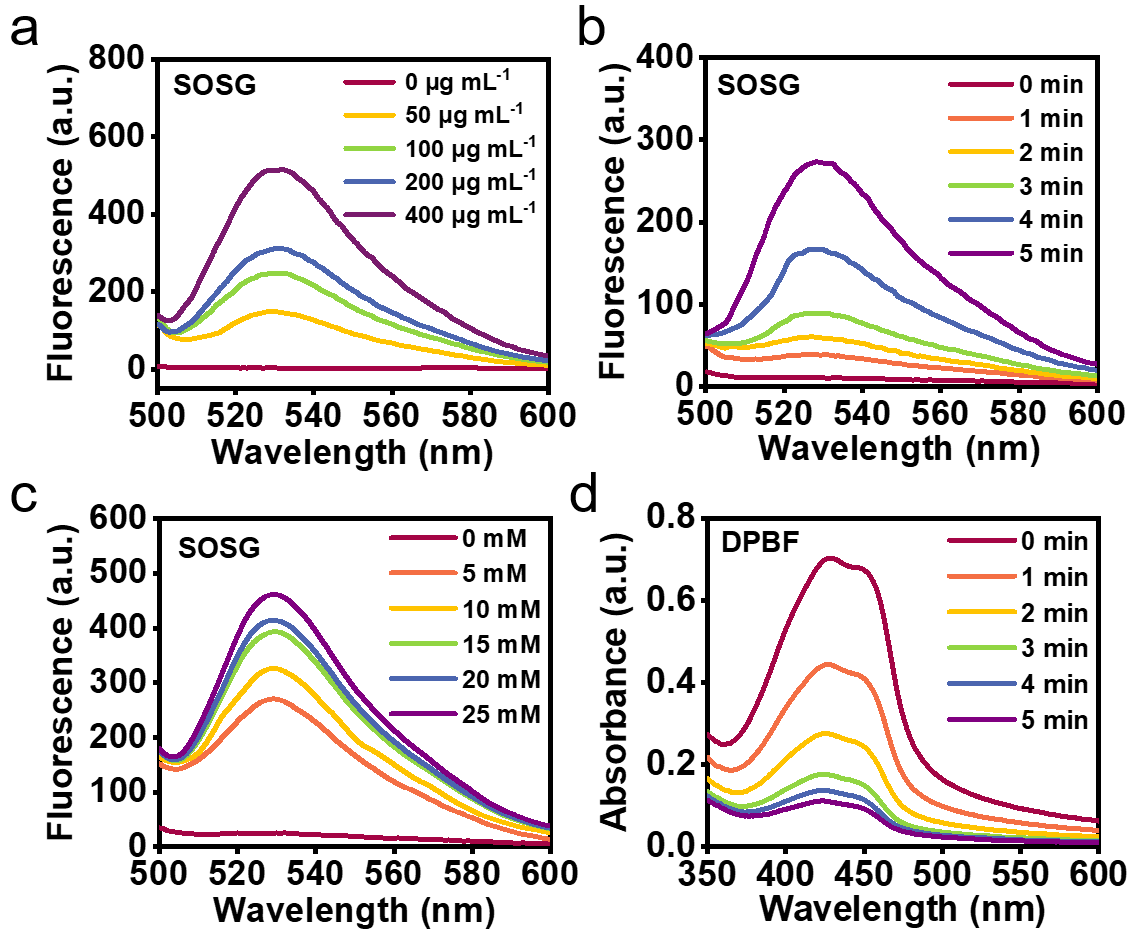


**Figure S13.** (a) Fluorescence spectra of SOSG (5 μM) containing CFA at different concentrations (0-400 μg mL^-1^) in the presence of H_2_O_2_ (10 mM) after US irradiation for (1.0 MHz, 1.0 W cm^-2^, 50% duty cycle) for 5 min (λ_ex_ = 488 nm). (b) Fluorescence spectra of SOSG (5 μM) containing CFA (200 μg mL^-1^) in the presence of H_2_O_2_ (10 mM) after US irradiation for (1.0 MHz, 1.0 W cm^-2^, 50% duty cycle) for a range of periods (0-5 min, λ_ex_ = 488 nm). (c) Fluorescence spectra of SOSG (5 μM) containing CFA (200 μg mL^-1^) in the presence of H_2_O_2_ at different doses (0-25 mM) after US irradiation for (1.0 MHz, 1.0 W cm^-2^, 50% duty cycle) for 5 min (λ_ex_ = 488 nm). (d) Absorbance decay of DPBF (10 μg mL^-1^) at 417 nm containing CFA (200 μg mL^-1^) in the presence of H_2_O_2_ (10 mM) after US irradiation for (1.0 MHz, 1.0 W cm^-2^, 50% duty cycle) for different periods (0-5 min).


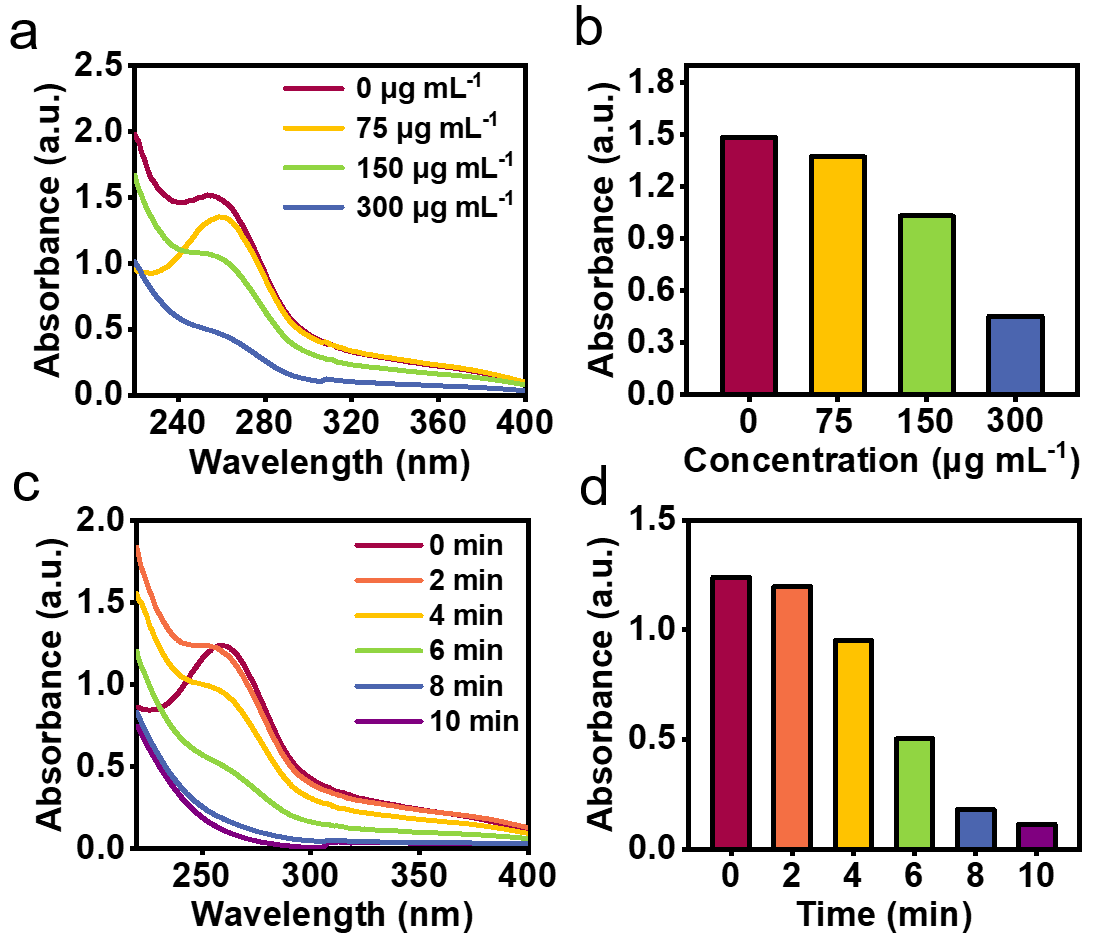


**Figure S14.** (a) Optical absorption spectra of NBT (25 μg mL^-1^) containing CFA at different concentrations (0-300 μg mL^-1^) in the presence of H_2_O_2_ (10 mM) after US irradiation for (1.0 MHz, 1.0 W cm^-2^, 50% duty cycle) for 5 min. (b) Absorption intensity of NBT at 260 nm corresponding to panel (a). (c) Optical absorption spectra of NBT (25 μg mL^-1^) containing CFA (200 μg mL^-1^) in the presence of H_2_O_2_ (10 mM) after US irradiation for (1.0 MHz, 1.0 W cm^-2^, 50% duty cycle) for different periods (0-10 min). (d) Absorption intensity of NBT at 260 nm corresponding to panel (c).


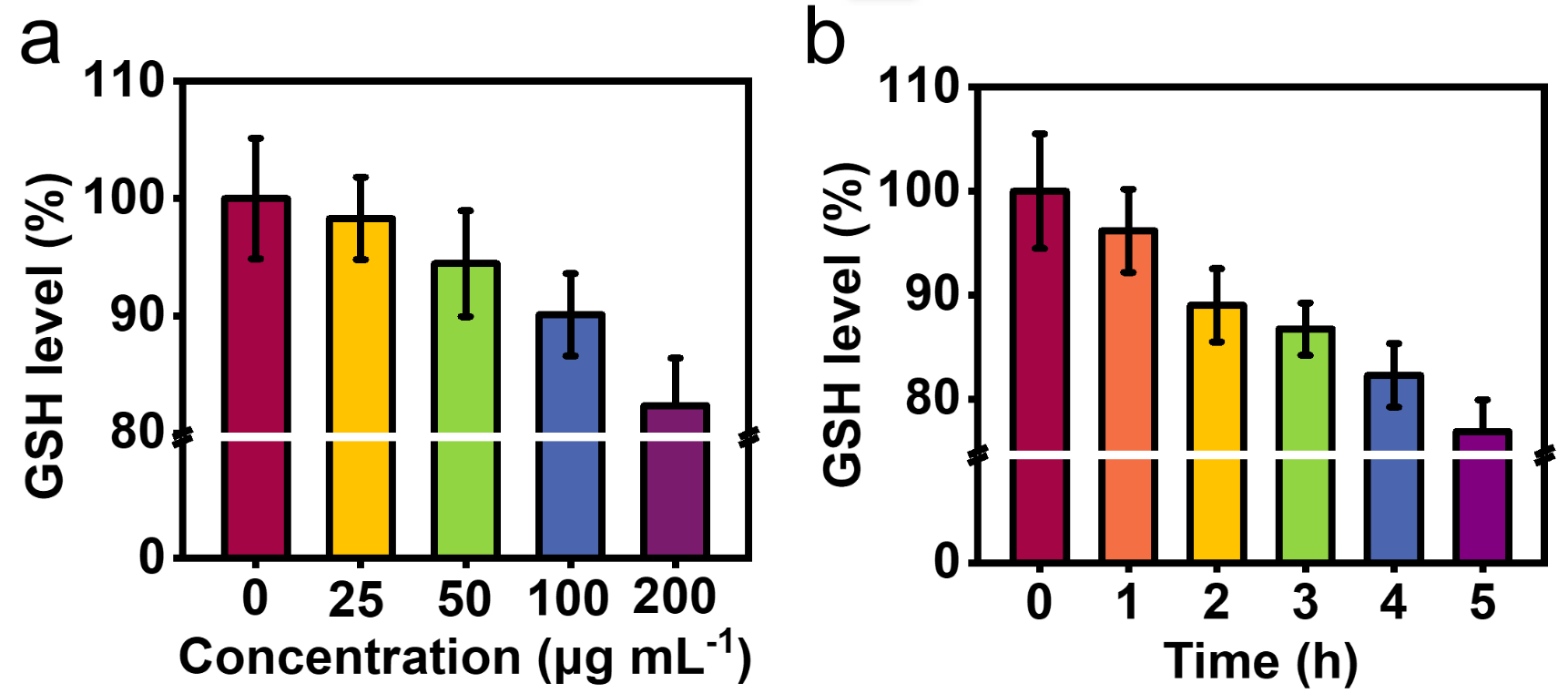


**Figure S15.** (a) Relative GSH level after incubation with CFA at different concentrations (0-200 μg mL^-1^) for 4 h. (b) Relative GSH level after incubation with CFA (200 μg mL^-1^) for diverse periods (0-5 h). Data are displayed as mean ± SD (n = 4).


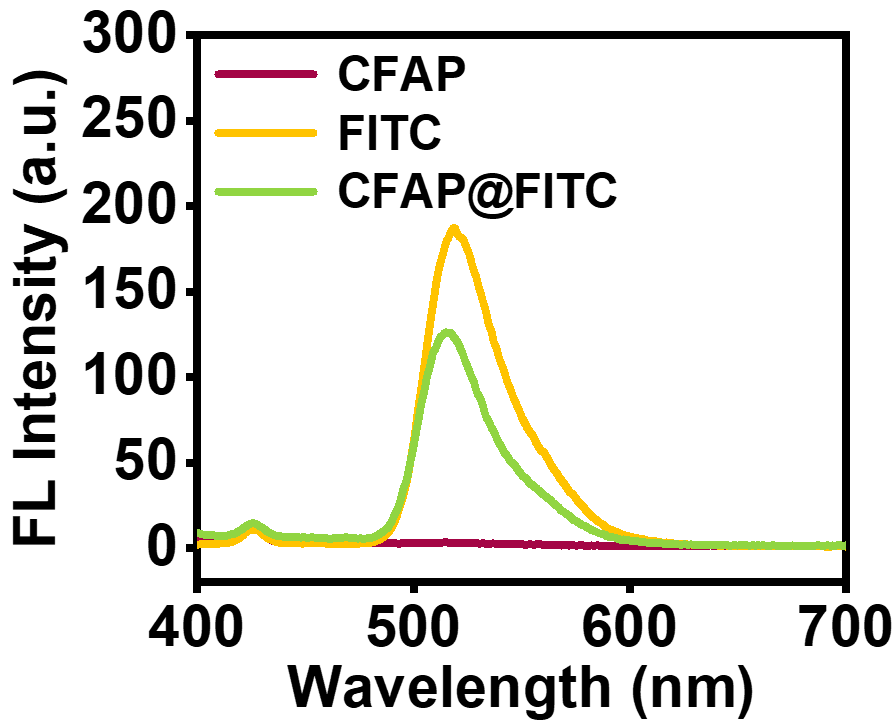


**Figure S16.** Fluorescence spectra of CFAP, FITC and CFAP@FITC (λ_ex_ = 480 nm).


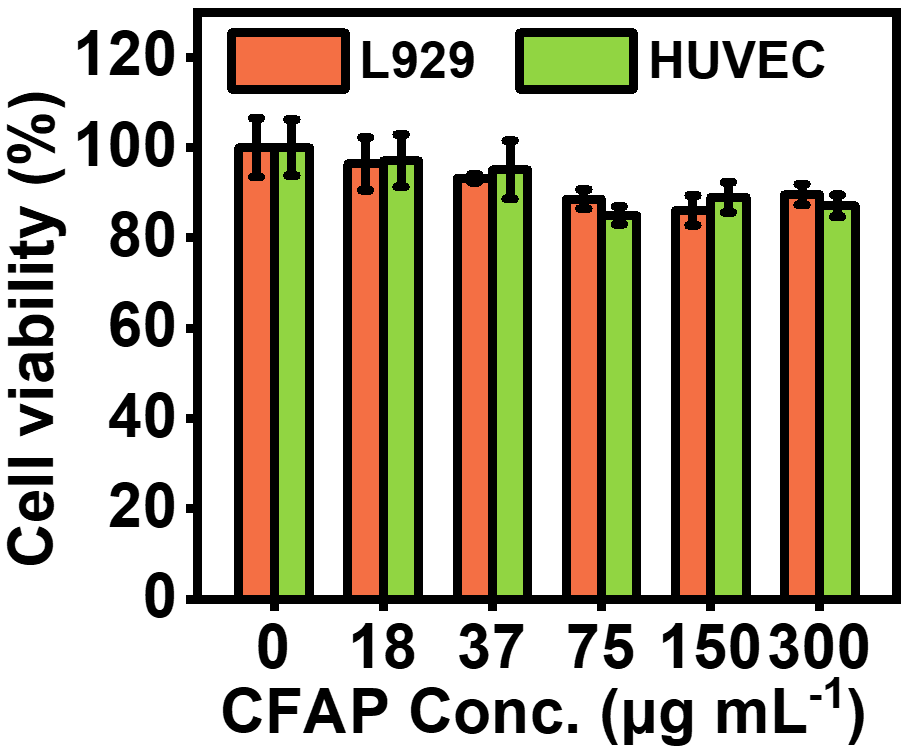


**Figure S17.** Viability of L929s and HUVECs after treatment with CFAP at different concentrations for 24 h. Data are displayed as mean ± SD (n = 4).


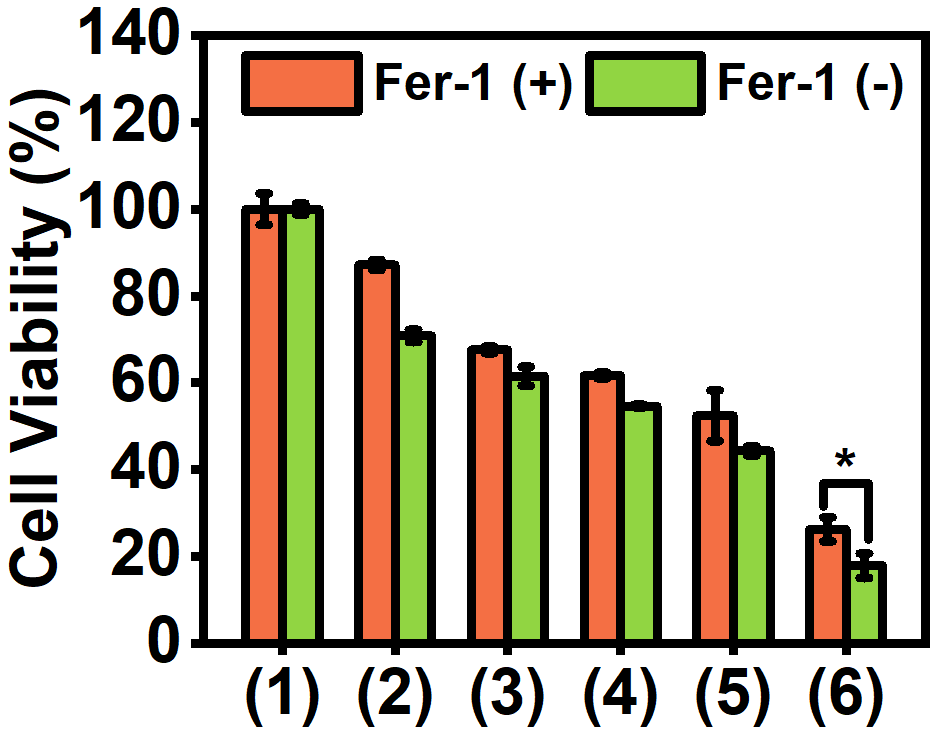


**Figure S18.** Viability of 4T1 cells after various treatments in the presence or absence of Fer-1. Groups are allocated to be (1) Blank, (2) US, (3) CFP, (4) CFAP, (5) CFP + US, (6) CFAP + US. Data are displayed as mean ± SD (n = 4). ^*^*p* < 0.05.


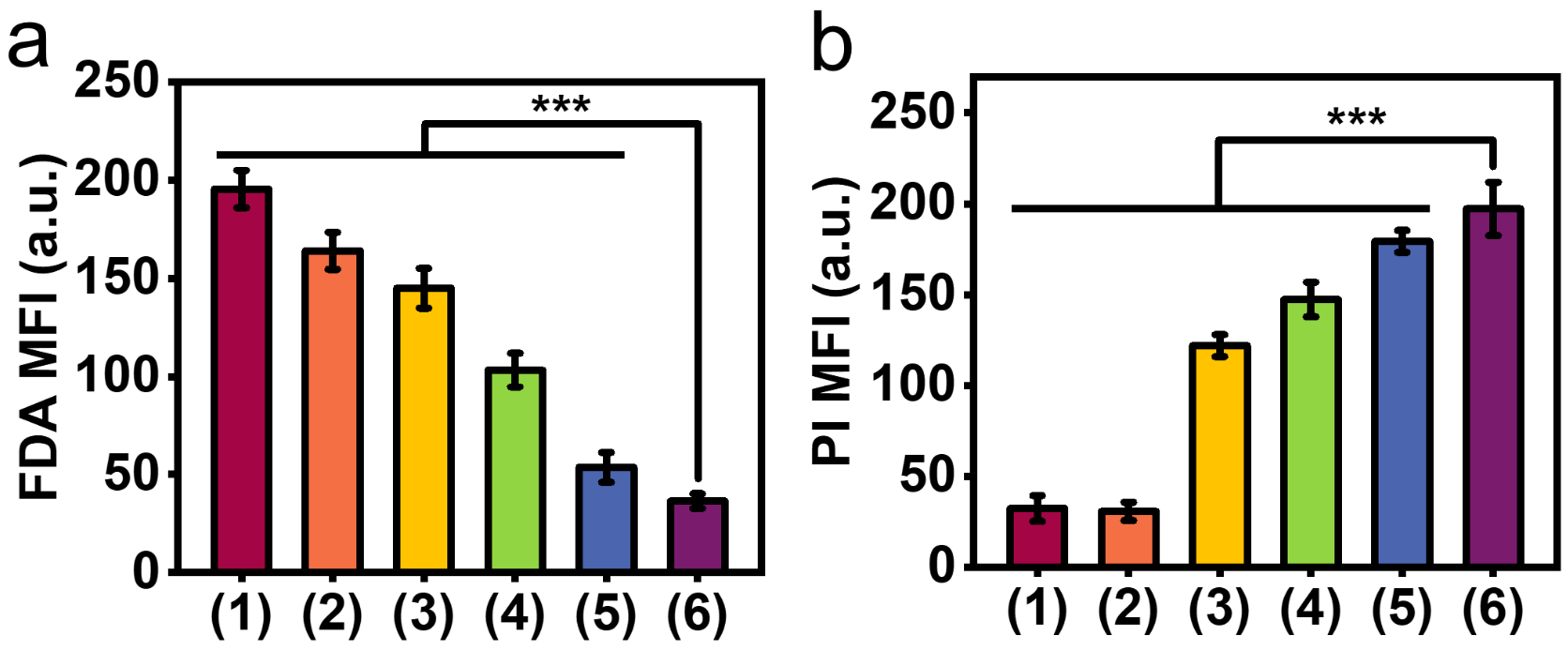


**Figure S19.** MFI of (a) FDA and (b) PI in terms of different groups corresponding to Figure 5c. Groups are allocated to be (1) Blank, (2) US, (3) CFP, (4) CFAP, (5) CFP + US, (6) CFAP + US. Data are displayed as mean ± SD (n = 4). ^***^*p* < 0.001.


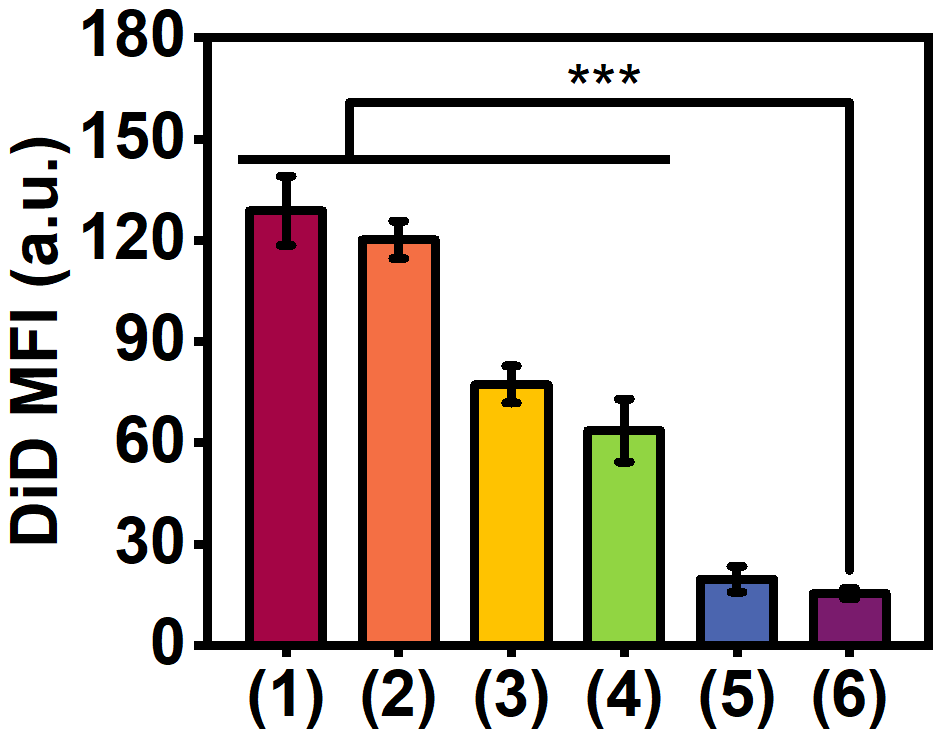


**Figure S20.** MFI of DiD in terms of different groups corresponding to Figure 5d. Groups are allocated to be (1) Blank, (2) US, (3) CFP, (4) CFAP, (5) CFP + US, (6) CFAP + US. Data are displayed as mean ± SD (n = 4). ^***^*p* < 0.001.


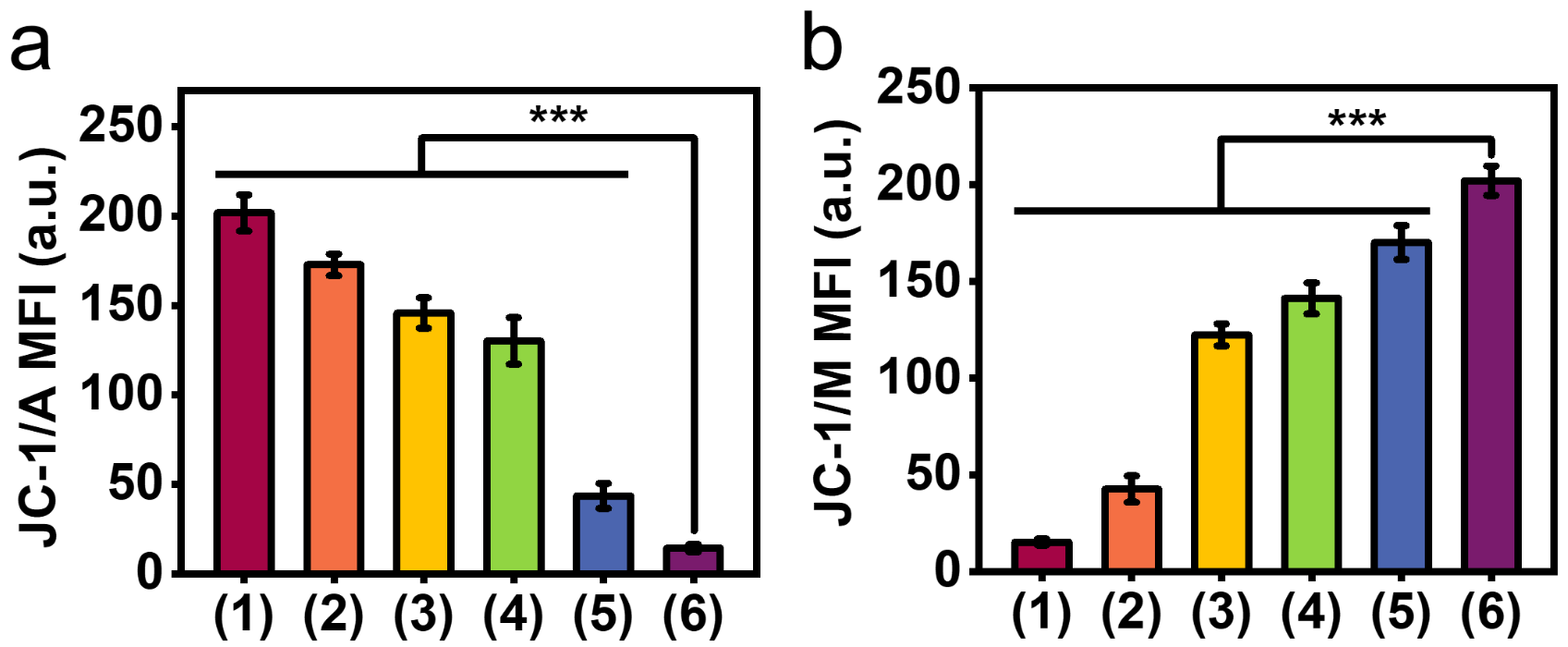


**Figure S21.** MFI of (a) JC-1/A and (b) JC-1/M corresponding to Figure 5e. Groups are allocated to be (1) Blank, (2) US, (3) CFP, (4) CFAP, (5) CFP + US, (6) CFAP + US. Data are displayed as mean ± SD (n = 4). ^***^*p* < 0.001.


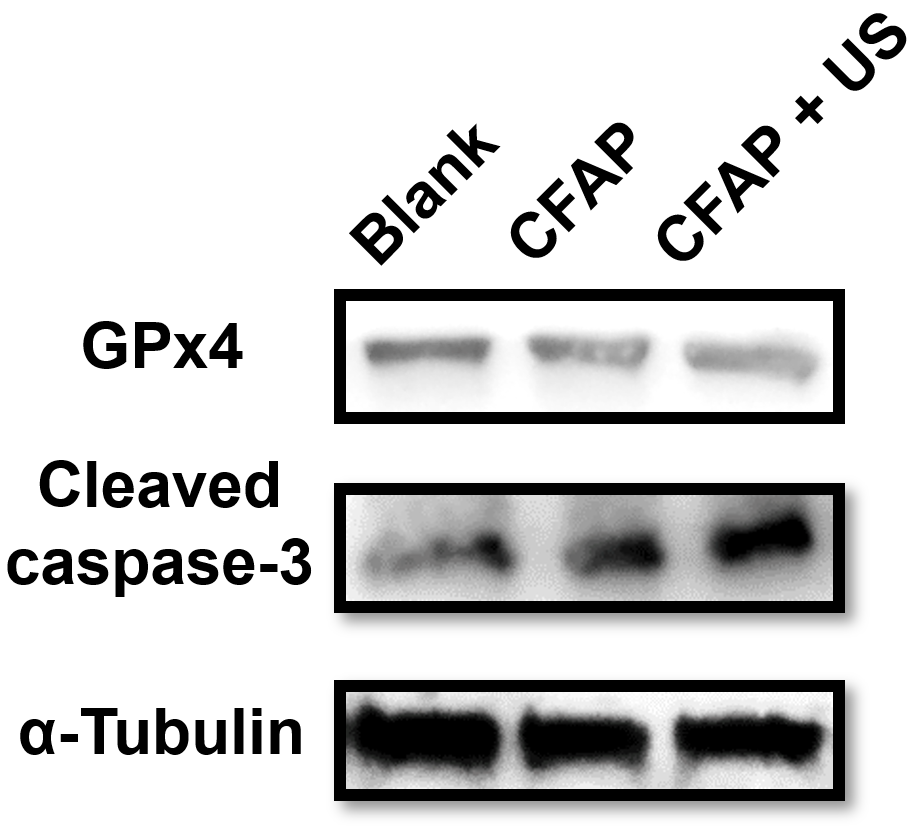


**Figure S22.** Western blot to analyze the expression levels of GPx4 and cleaved caspase-3 in cytosolic region after different treatments.


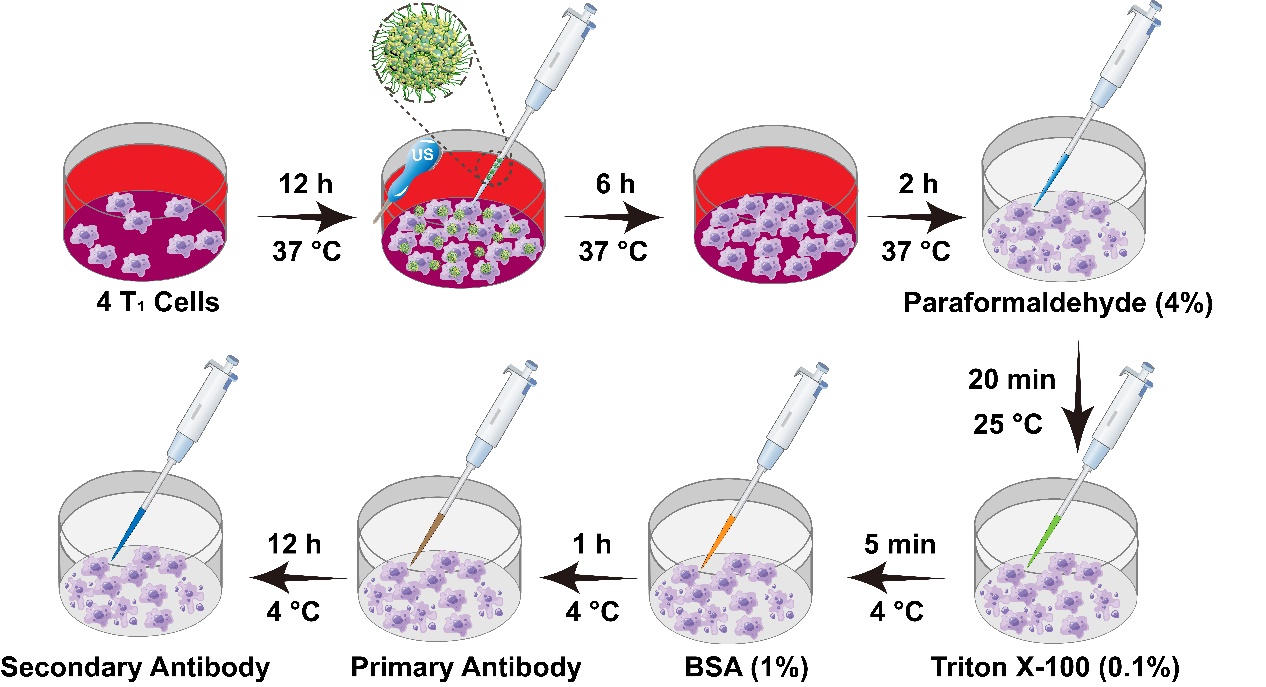


**Figure S23.** Schematic illustration of an optimized protocol of immunostaining to evaluate the CRT exposure and HMGB1 release from tumor cells.


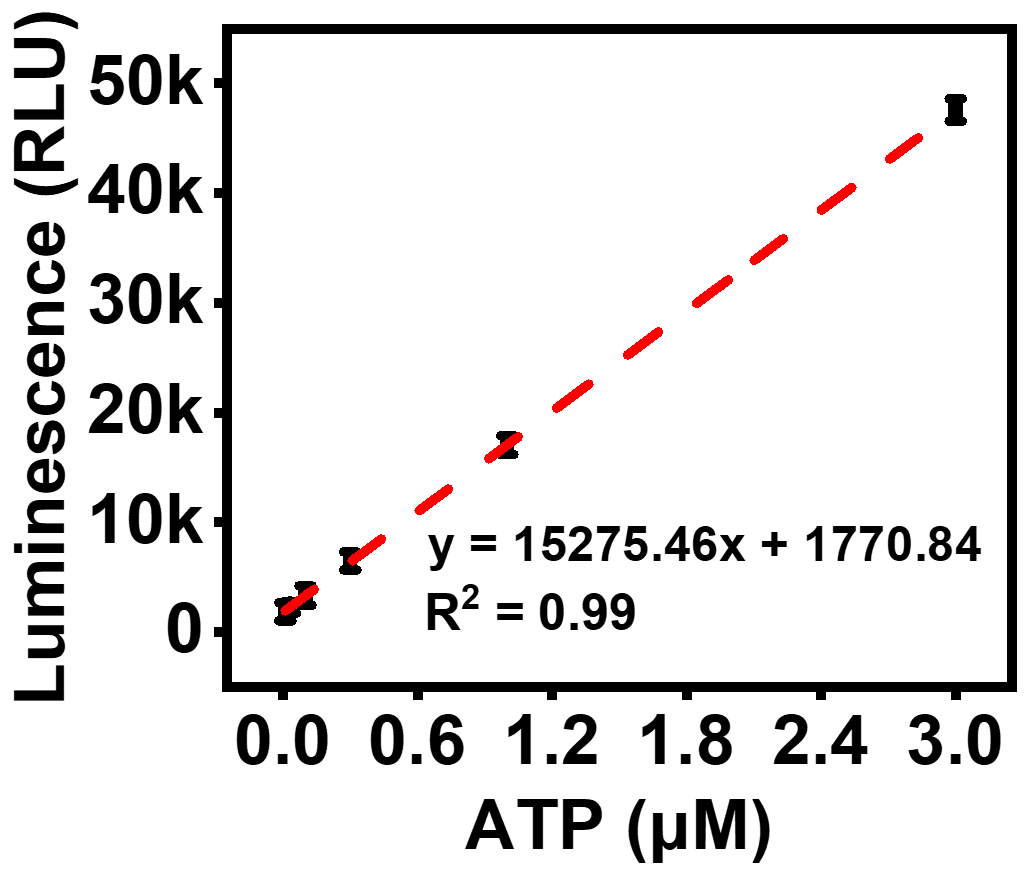


**Figure S24.** Calibration curve of luminescence *vs.* ATP concentration from ATP assay kit.


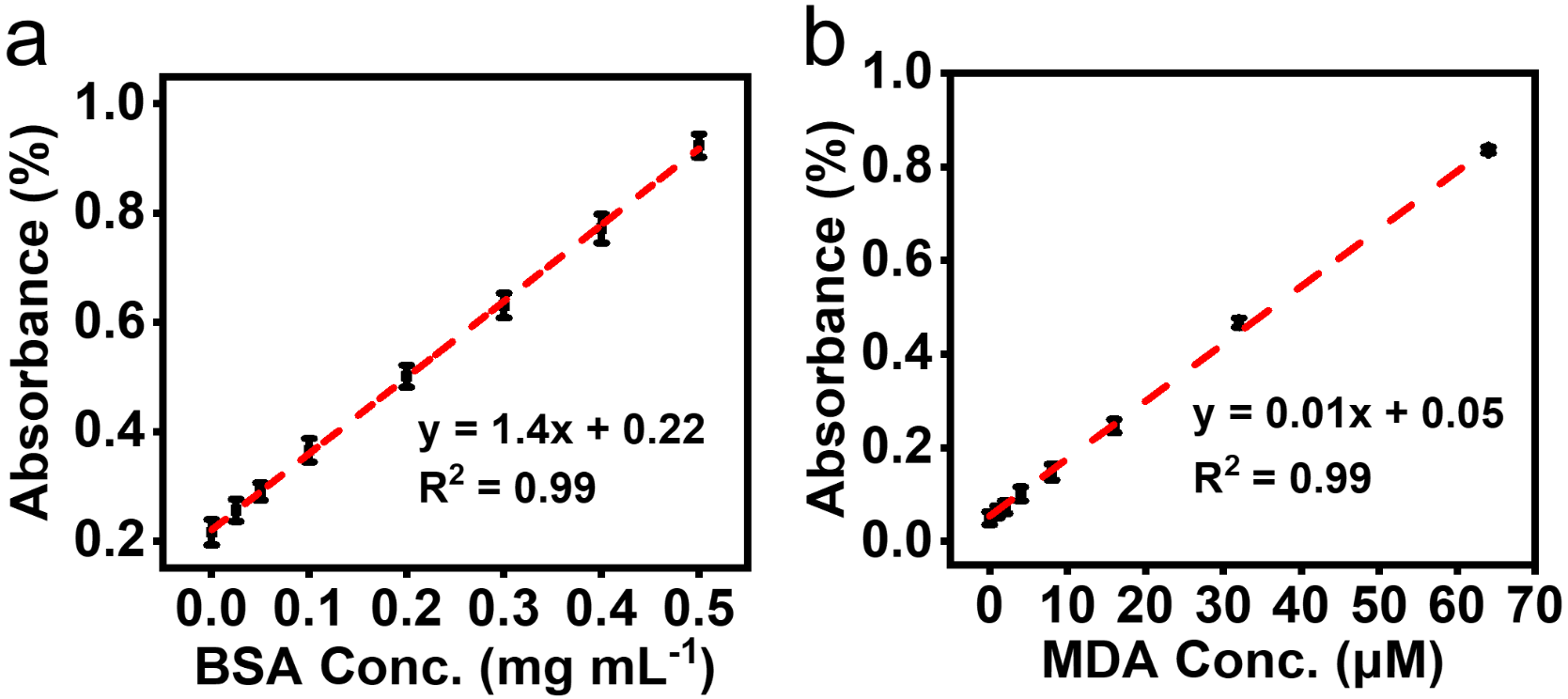


**Figure S25.** Calibration curves of BCA protein quantitative kit (562 nm absorbance *vs.* BSA concentration) and lipid peroxidation (MDA) assay kit (532 nm absorbance *vs.* MDA concentration).


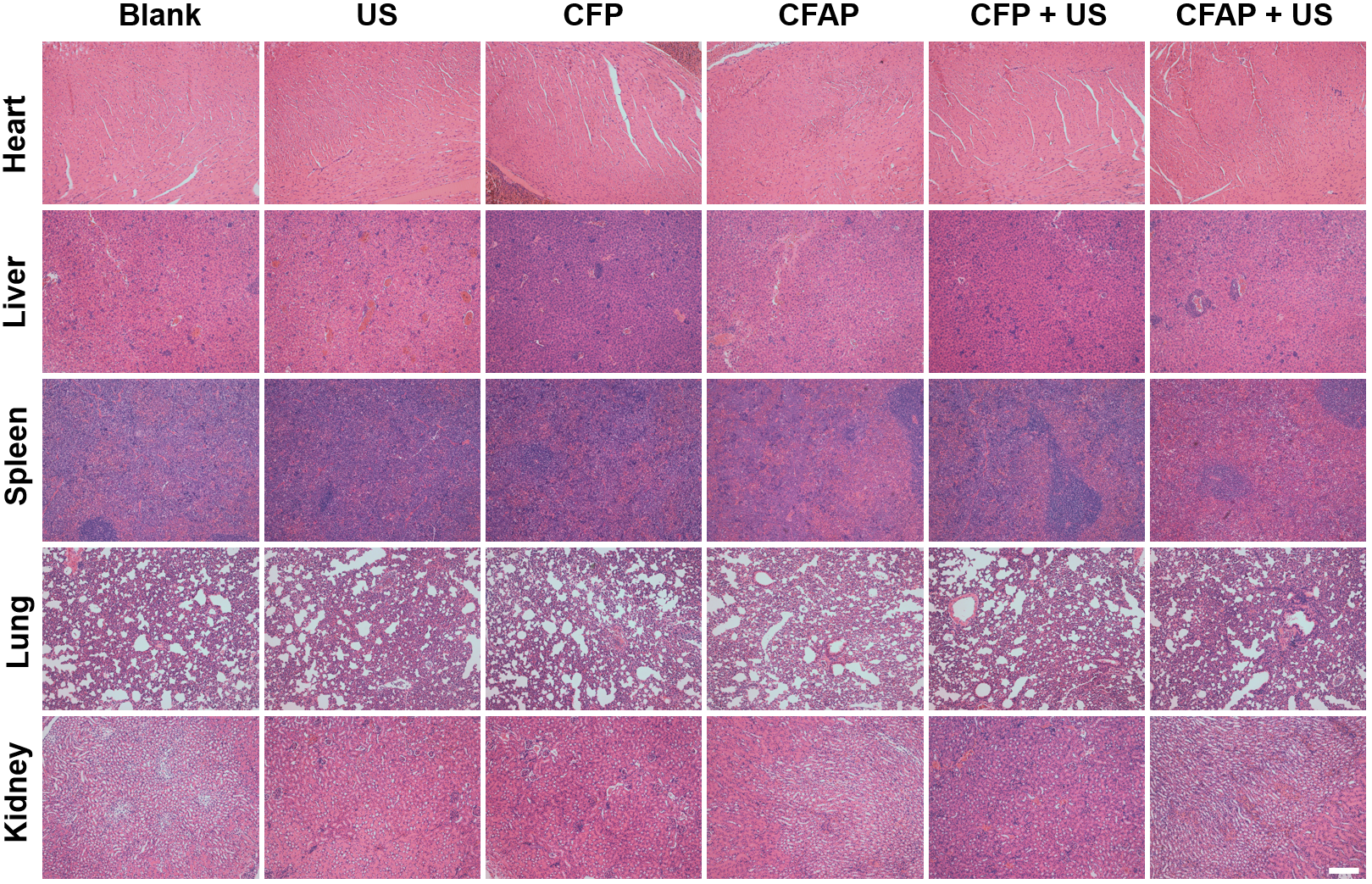


**Figure S26.** Histopathological analysis of major organ sections on day 14 through H&E staining (scale bar: 100 μm).


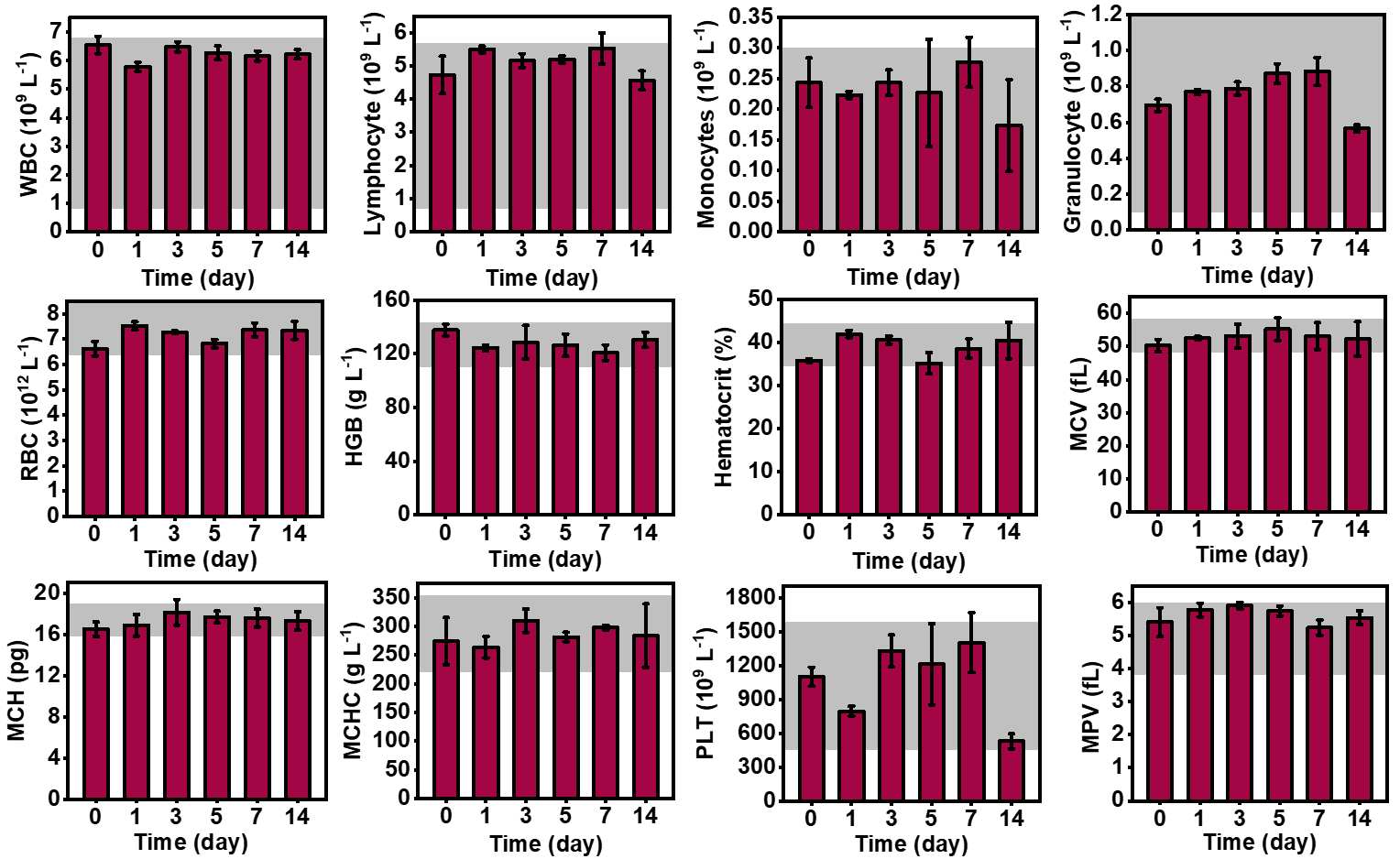


**Figure S27.** Key indexes of blood routine examination after BALB/c mice being intravenously injected with CFAP (25 mg kg^-1^, in saline), including including white blood cells (WBC), lymphocytes (LY), monocytes (MNC), red blood cells (RBC), hemoglobin (HGB), granulocytes (GR), platelets (PLT), mean corpuscular volume (MCV), hematocrit (HCT), mean corpuscular hemoglobin (MCH), mean platelet volume (MPV) and mean corpuscular hemoglobin concentration (MCHC). Data are displayed as mean ± SD (n = 4).


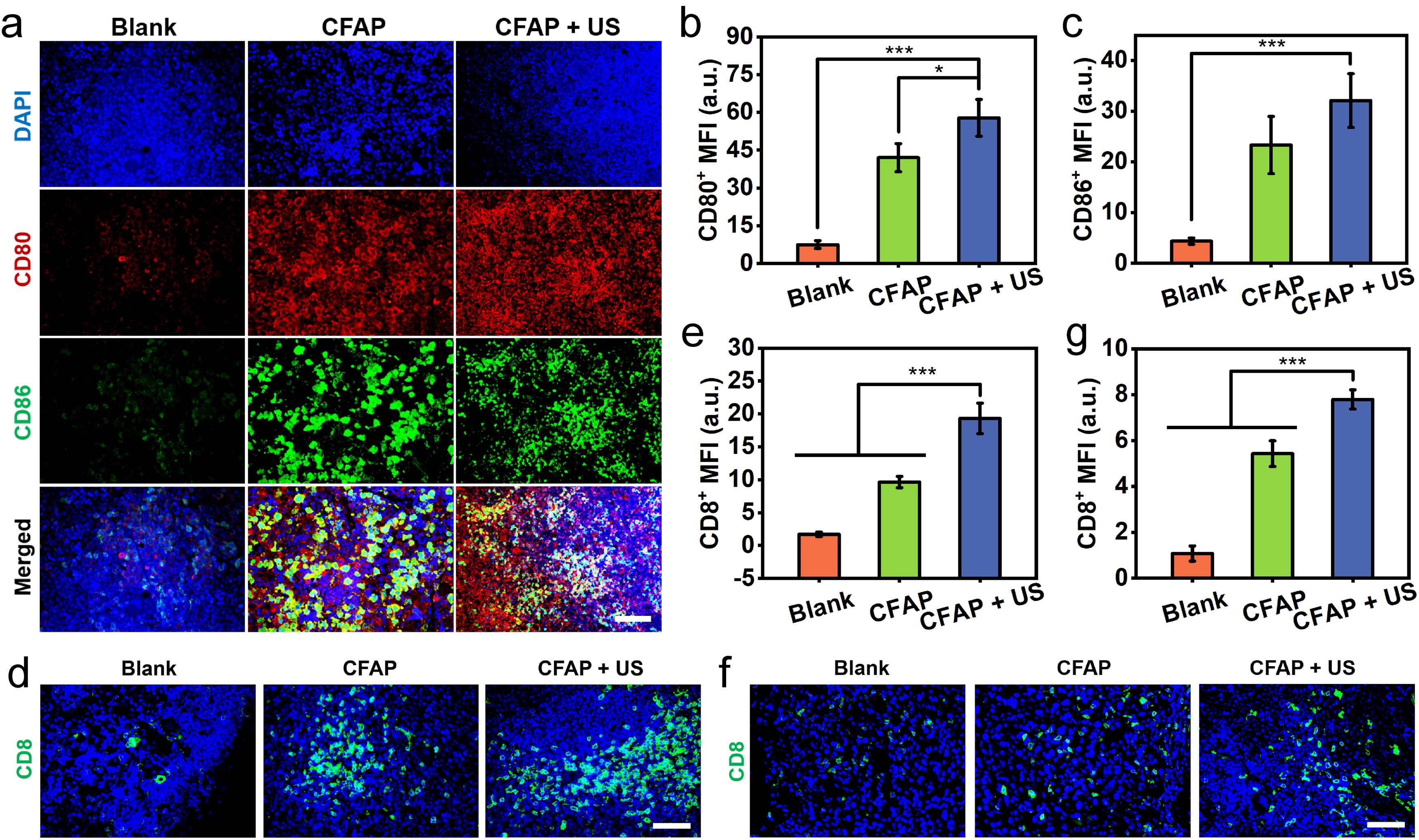


**Figure S28.** Antitumor immunity stimulation on 4T1 tumor-bearing BALB/c mice. (a) Confocal microscopy of spleen sections after immunofluorescence staining with CD80 and CD86 in terms of different therapeutic regimens (scale bar: 50 µm). MFI of (b) CD80 and (c) CD86 corresponding to panel (a). (d) Confocal microscopy of spleen sections after immunofluorescence staining with CD8 (scale bar: 50 µm). (e) MFI of CD8 corresponding to panel (d). (f) Confocal microscopy of tumor sections after immunofluorescence staining with CD8 (scale bar: 50 µm). (g) MFI of CD8 corresponding to panel (f). Data are displayed as mean ± SD (n = 5). ^*^*p* < 0.05, ^***^*p* < 0.001.


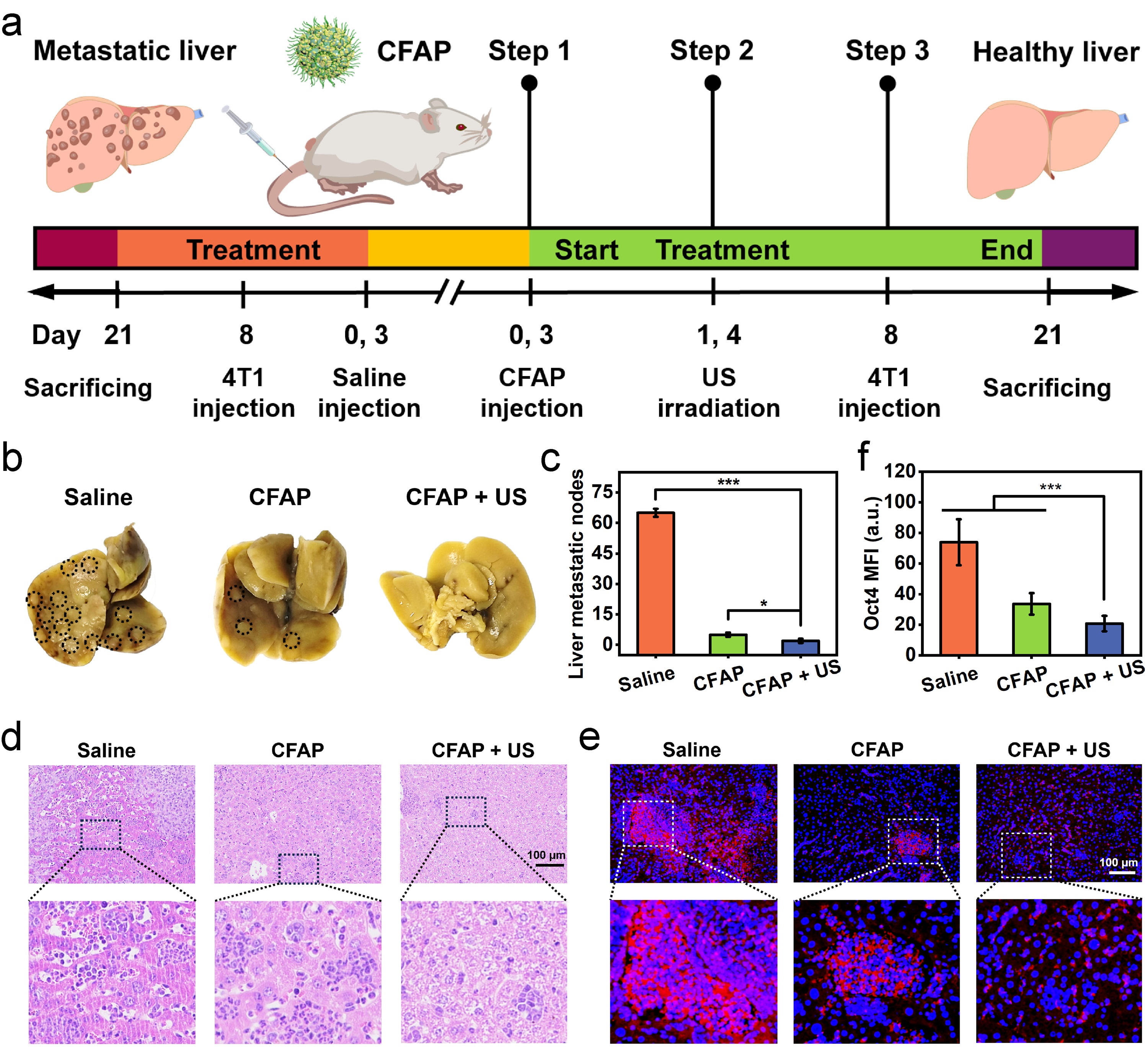


**Figure S29.** Anti-metastasis *in vivo*. (a) Procedure chart to display the therapeutic regimen of hepatic metastasis on BALB/c mice-bearing 4T1 tumors. (b) Representative photos of hepatic metastatic nodules on day 21. (c) Average number of metastatic nodules on the liver corresponding to panel (b). (d) Microscopic images of liver tissue sections with H&E staining on day 21. (e) Fluorescence images of liver tissue sections after immunofluorescence staining with Oct4. (f) MFI of Oct4 corresponding to panel (e). Data are displayed as mean ± SD (n = 5). ^*^*p* < 0.05, ^***^*p* < 0.001.
